# EEG Correlates of Learning From Speech Presented in Environmental Noise

**DOI:** 10.3389/fpsyg.2020.01850

**Published:** 2020-11-05

**Authors:** Ehsan Eqlimi, Annelies Bockstael, Bert De Coensel, Marc Schönwiesner, Durk Talsma, Dick Botteldooren

**Affiliations:** ^1^WAVES Research Group, Department of Information Technology, Ghent University, Ghent, Belgium; ^2^École d'Orthophonie et d'Audiologie, Université de Montréal, Montreal, QC, Canada; ^3^Erasmushogeschool Brussel, Brussels, Belgium; ^4^ASAsense, Bruges, Belgium; ^5^Faculty of Biosciences, Pharmacy and Psychology, Institute of Biology, University of Leipzig, Leipzig, Germany; ^6^International Laboratory for Brain, Music and Sound Research (BRAMS), Université de Montréal, Montreal, QC, Canada; ^7^Department of Experimental Psychology, Ghent University, Ghent, Belgium

**Keywords:** auditory attention, auditory perception, EEG, inhibition, learning context, long-range temporal correlations, speech in noise, speech processing

## Abstract

How the human brain retains relevant vocal information while suppressing irrelevant sounds is one of the ongoing challenges in cognitive neuroscience. Knowledge of the underlying mechanisms of this ability can be used to identify whether a person is distracted during listening to a target speech, especially in a learning context. This paper investigates the neural correlates of learning from the speech presented in a noisy environment using an ecologically valid learning context and electroencephalography (EEG). To this end, the following listening tasks were performed while 64-channel EEG signals were recorded: (1) attentive listening to the lectures in background sound, (2) attentive listening to the background sound presented alone, and (3) inattentive listening to the background sound. For the first task, 13 lectures of 5 min in length embedded in different types of realistic background noise were presented to participants who were asked to focus on the lectures. As background noise, multi-talker babble, continuous highway, and fluctuating traffic sounds were used. After the second task, a written exam was taken to quantify the amount of information that participants have acquired and retained from the lectures. In addition to various power spectrum-based EEG features in different frequency bands, the peak frequency and long-range temporal correlations (LRTC) of alpha-band activity were estimated. To reduce these dimensions, a principal component analysis (PCA) was applied to the different listening conditions resulting in the feature combinations that discriminate most between listening conditions and persons. Linear mixed-effect modeling was used to explain the origin of extracted principal components, showing their dependence on listening condition and type of background sound. Following this unsupervised step, a supervised analysis was performed to explain the link between the exam results and the EEG principal component scores using both linear fixed and mixed-effect modeling. Results suggest that the ability to learn from the speech presented in environmental noise can be predicted by the several components over the specific brain regions better than by knowing the background noise type. These components were linked to deterioration in attention, speech envelope following, decreased focusing during listening, cognitive prediction error, and specific inhibition mechanisms.

## 1. Introduction

The human brain is remarkably capable of focusing on one specific sound while suppressing all others (Alain, 2007). Nevertheless, processing of relevant information largely depends on the specific interaction of the acoustic features of speech and noise signals, their informative content, attention, state, and the prior knowledge (familiarity with the presented topic) of the listener (Szalma and Hancock, 2011). To understand the underlying mechanisms of this diverse phenomenology in human sound interaction, short-term features of distracting events, state of the listener, information flow, and loss of efficiency need to be studied. One key aspect of the study design is ecological validity (Chaytor and Schmitter-Edgecombe, 2003), meaning that realistically complex stimuli and conditions are included possibly in addition to artificially designed stimuli.

In a learning context, the ability to acquire and retain vocal information strongly affects the overall learning performance. This is even more challenging when this occurs in the presence of environmental noise. One of the effects involved in this ability is known as the cocktail party effect (Cherry, 1978), and this refers to the ability of the brain to direct attention to a target sound despite the presence of distracting sounds. Although the underlying mechanisms are indispensable to learn from information presented in an acoustically rich environment (Lehmann and Schönwiesner, 2014), they are far from fully understood.

Attention directs both cognitive and sensory resources to the target sounds (Schneider and Shiffrin, 1977). In general, such resources are limited in capacity based on the bottleneck (Pashler, 1984) and capacity sharing (Kahneman, 1973) theories. Most of the observed effects of noise on learning (Alain, 2007) can be explained by attention, including unlocking undesired attention focus as well as an increased cognitive load when listening to speech in noise (Rudner, 2016). Moreover, listening performance and speech intelligibility in background noise can be impaired by distracting attention away from the narrative and hampering relevant sounds (Ljung et al., 2009; Clark and Sörqvist, 2012). However, attention focusing and appropriate gating of (ir)relevant stimuli are not only the matter of cortical processing but also peripheral neurophysiological stages of auditory analysis are involved. Attention can be modulated by bottom-up factors (referring to external stimulus-driven responses that guide the attention due to inherent properties of salient events relative to the background) as well as top-down task-specific functions (referring to internal modulation of attention that is driven by cognition based on prior knowledge, expectations, and learned schemas) (Katsuki and Constantinidis, 2014; Kaya and Elhilali, 2017).

Auditory attention-related research (especially bottom-up attention) mostly adopts an event-related potential (ERP) design (Alain, 2007). However, a classical ERP design with repeated stimuli conflicts with the idea of ecologically valid stimuli and studying top-down attention. In the current paper, the single-trial EEG experiment was used to study how auditory-related neural responses vary depending on acoustical stimulus and listening condition. The power spectrum of EEG signal exhibits peaks in different frequency ranges reflecting different underlying mechanisms (Buzsáki et al., 2013; He, 2014). Therefore, one of the most common methods to process the single-trial EEG signals is spectral analysis, which relies on partitioning the signal into the different frequency sub-bands (Clayton et al., 2015).

Previous studies using spectral analysis have shown different frequency bands contribute to the various underlying mechanisms during listening to speech in noise, such as top-down attention (Gazzaley and Nobre, 2012), cortical inhibition (Uusberg et al., 2013), language processing (Pulvermüller et al., 1997), neural entrainment to speech (Riecke et al., 2018), and excitation-inhibition balance (Poil et al., 2012). The roles of the different frequency bands in these mechanisms are discussed separately below.

Low-frequency EEG signals (1 − 8 Hz) can be modulated by attention (Kerlin et al., 2010; Braboszcz and Delorme, 2011). Two important mechanisms may be associated with the low-frequency EEG. The first one is the mismatch between current and desired levels of attention (Clayton et al., 2015) and the transition of the fatigue state (Borghini et al., 2014), which is observed as a continuous increase of low-frequency power with time on task [unlike the alpha-band activity (8 − 13 Hz) (Mierau et al., 2017)]. Frontomedial theta-band (4 − 8 Hz) activity has been linked to both enhanced attention over short time-scale cognitive tasks and reduced attention (increased attentional fatigue) following long time-scale cognitive tasks (Wascher et al., 2014; Clayton et al., 2015). Moreover, it has been shown that the delta-band (1 − 4 Hz) absolute power is higher in the mind wandering compared to the focused state over the fronto-central region (Braboszcz and Delorme, 2011).

The second mechanism is the information and attention selection (Schroeder and Lakatos, 2009; Herrmann et al., 2016). This means that the attention can use a mechanism of selection leading to oscillatory entrainment to a task-relevant stimulus (Schroeder and Lakatos, 2009). However, neural entrainment is a broader concept and refers to the temporal alignment of neural signals with regularities in an exogenously occurring stimulus, such as speech (Obleser and Kayser, 2019) and even aperiodic (speech) signals (Obleser et al., 2012; Goswami and Leong, 2013).

Speech following (and speech envelope following/tracking) as one the manifestation of the neural entrainment refers to the relation between the neural and sound signals (Obleser and Kayser, 2019). Although it has been measured in various frequency bands (Obleser and Kayser, 2019), its impact on low-frequency EEG (delta and theta bands) has been shown in several electrophysiological experiments (Luo and Poeppel, 2007; Doelling et al., 2014; O'Sullivan et al., 2014; Kayser et al., 2015). The basic hypotheses of these studies are the following: (1) entrainment occurs also at other frequencies, but this effect is obscured by stronger signals; (2) the low-frequency speech envelope entrainment of brain activity could be robust against different background noises (Ding et al., 2014); and (3) the speech envelope is constituted by slow temporal modulations, which contribute to speech recognition despite different background sounds (Houtgast and Steeneken, 1985; Rosen, 1992; Kerlin et al., 2010; Ding and Simon, 2013; Ríos-López et al., 2017).

It has also been shown that attended and unattended stimuli could be decoded by low-frequency single-trial EEG in a cocktail party scenario based on a stimulus-reconstruction algorithm (O'Sullivan et al., 2014). This stimulus-reconstruction method indicated the slow amplitude envelope of attended speech (≤ 8 Hz) is tracked more strongly by the low-frequency EEG (2−8 Hz) compared to the unattended speech. Furthermore, it has been shown that in the multi-talker speech perception, the attended speaker is represented over the non-primary auditory cortex (AC) while the individual speakers are represented over the primary AC (O'Sullivan et al., 2019).

Alpha-band activity (~8 − 13 Hz) is also often modulated by auditory attention, especially by the inhibition function (Strauß et al., 2014). The term “alpha-as-inhibition” is used to highlight that alpha-band activity, beyond resting state, could reflect inhibition of the distracting sound (Clark, 1996; Uusberg et al., 2013). Increased alpha-band activity over the task-irrelevant brain regions reflects less involvement of those regions. Hence, comparison of alpha power between task-relevant and task-irrelevant cortical regions can be an indicator for inhibition (Pfurtscheller and Da Silva, 1999; Chang et al., 2010). In fact, alpha event-related synchronization (ERS) reflects inhibition and alpha event-related desynchronization (ERD) releases from inhibition (Foxe et al., 1998; Snyder and Foxe, 2010; Klimesch, 2012).

Not only absolute alpha power over a fixed frequency band but also alpha peak frequency (APF) and its corresponding power can be associated with attention, inhibition, memory, and cognitive demand (Klimesch, 1997; Clark et al., 2004; Haegens et al., 2014; Gulbinaite et al., 2017). APF (Doppelmayr et al., 1998) and individual alpha frequency (IAF) (Klimesch, 1999) indicate the actual frequency limits of alpha activity, which exhibit variability within and between subjects (Haegens et al., 2014). APF is also linked to the number of spiking neurons or the input level (Mierau et al., 2017). If the input level increases with respect to the baseline level, APF increases until the oscillation becomes unstable and then it is replaced by a lower frequency (Mierau et al., 2017). Although APF increases with a higher allocation of attentional resources, it decreases with lower attentional demand and cognitive load due to unstable state and overloaded attention capacity (Hutt et al., 2016; Mierau et al., 2017). Higher APF can be accompanied by lower alpha power resulting in task-relevant regions that exhibit increased APF during task performance (Hutt et al., 2016). Studies focusing on power-related frequency shifts have suggested a rather complex relationship between alpha frequency and power (Kawabata, 1972). Other studies have shown that APF decreases with increasing attentional demand and task difficulty (Angelakis et al., 2004; Haegens et al., 2014), which could be explained by unstable state and overloaded attention capacity. Enhanced APF might reflect a state of cognitive preparedness and the attentional switch between wandering and focused states of mind (Braboszcz and Delorme, 2011).

In addition to the peaks at the frequency ranges, a predominant “$\frac{1}{f}$” component in the EEG power spectra leads a power-law function, i.e., $p\propto {\frac{1}{f}}^{a}$, where *p* is power, *f* is frequency, and *a* is the scaling exponent (He, 2014). Therefore, the EEG time series exhibit scale-free dynamics and do not have a characteristic scale (He et al., 2010; He, 2014). Furthermore, the ongoing EEG signals hold a memory of their own dynamics on time-scales, which could be linked to the scale-free dynamics and the self-similarity concept in fractal geometry (Palva et al., 2013). Long-range temporal correlations (LRTC) are the most common measures with which to quantify how slowly the autocorrelations of the signal decay in power-law function (Linkenkaer-Hansen et al., 2001; Nikulin and Brismar, 2005; Palva et al., 2013). Alpha-band LRTC could reflect an optimal balance between excitation and inhibition states (Poil et al., 2012). Decreased alpha-band LRTC compared to the resting state correlates with better attentional performance (Colosio et al., 2017). Higher alpha-band LRTC during resting-state could predict high performance in decision making (Colosio et al., 2017), working memory (Mahjoory et al., 2019) and attention tasks (Irrmischer et al., 2018).

Increased beta-band (~13 − 30 Hz) power over the fronto-lateral region has been observed in the mind wandering compared to focused state (Braboszcz and Delorme, 2011). Furthermore, the beta-band activity can be related to the maintenance of current sensorimotor or cognitive task (Engel and Fries, 2010; Weiss and Mueller, 2012; Zhao et al., 2012). A quasi-harmonic relationship has been suggested between the beta and alpha peaks or central frequencies only during rest (Van Albada and Robinson, 2013; Haegens et al., 2014). The lack of a strict relationship between the beta and alpha peak frequencies during task-based conditions may reflect independent networks being activated (Jones et al., 2009; Haegens et al., 2014).

Localized gamma-band activity (~30 − 45 Hz) has been found in task-relevant cortical regions (MacDonald and Barth, 1995; Cervenka et al., 2011; Siegel et al., 2011). Gamma-band activity plays a central role in attention, perception and language processing (Pulvermüller et al., 1997). Furthermore, gamma-band activity in sensory cortices has often been linked with enhanced attention to these particular sensory inputs (Ahveninen et al., 2013). It has also been shown that gamma-band power in auditory areas increases during extended auditory attention tasks (Kaiser and Lutzenberger, 2005; Ahveninen et al., 2013). According to popular theory, gamma waves may be implicated in creating the unity of conscious perception and semantic processing (Buzsaki, 2006).

In this study, we aimed to investigate the different mechanisms involved in learning from the speech presented in noise using single-trial EEG and mimicking an ecologically valid context. To this end, 23 participants were exposed to the following listening tasks while 64-channel EEG signals were recorded: (1) attending to a lecture in the background noise (LA), (2) attending to the background noise alone (BA), and (3) not attending to the sound while still being exposed to the background noise (BUA). For the background noise, realistic environmental sound fragments from continuous highway noise (HW), fluctuating traffic (FT), and multi-talker babble (MT) were used. A written exam on the lecture was taken after 13 sets of 5-min lectures and the BA task for assessing the amount of information that participants have actually acquired and retained from the lectures.

We hypothesized several neural mechanisms, such as cortical inhibition, auditory attention, neural entertainment, and predictive coding, can be affected by the listening conditions we have designed. Therefore, five qualitative hypotheses were considered: (1) alpha-as-inhibition, (2) excitation-inhibition balance reflected in the alpha band, (3) low-frequency envelope following, (4) maintenance of current cognitive task, and (5) semantic processing and cognitive prediction violation or error.

The alpha-as-inhibition hypothesis (Uusberg et al., 2013) implies that alpha-band activity mediates inhibition of task-irrelevant cortical areas. The excitation-inhibition balance hypothesis (Poil et al., 2012) relates the task performance and optimal information processing to the long-range temporal correlations of alpha-band activity. The low-frequency envelope following hypothesis (Luo and Poeppel, 2007; Kerlin et al., 2010; Obleser and Kayser, 2019) implies the neural entrainment and tracking of speech (and background sound) envelope can be reflected in the low-frequency bands, i.e., delta and theta frequency bands. However, here, the relation between the EEG and sound signals has not been analyzed (which is the main tool to measure the envelope following) due to our unsupervised approach. In fact, we have assumed that the strong representation of low-frequency EEG signals (i.e., changes in spectral characteristics) may be related to the envelope following. Although, the neural entrainment and envelope following occurs also at higher frequencies but this effect is obscured by stronger signals (note that no source reconstruction was used in this paper). The hypothesis of maintenance of current cognitive task (Spitzer and Haegens, 2017) implies that the preservation of the current brain state and the long-range communication can be associated with the beta-band activity. Finally, the last hypothesis suggests that semantic or higher-level processes (specifically semantic violations) due to speech processing induce power changes in the gamma-band activity (Braeutigam et al., 2001; Buzsaki, 2006; Hald et al., 2006; Penolazzi et al., 2009). Moreover, the generative models for the perception, such as the predictive coding (Sedley et al., 2016) assume the precision of prediction, changes to predictions, and violations (errors) in predictions are encoded with the alpha, beta, and gamma frequency bands, respectively. These assumptions can be in accordance with our hypotheses.

Since a few EEG indicators, such as alpha peak frequency and power, alpha long-range temporal correlations, and delta absolute power were evaluated in a recent work by our group (Eqlimi et al., 2019), a wider range of EEG features (see below) was estimated for investigating our hypotheses. More precisely, the following features were estimated: spectral features and peak frequency of the alpha-band activity (hypothesis 1), the alpha-band LRTC (hypothesis 2), the spectral features of the delta and theta (hypothesis 3), the beta (hypothesis 4), and the gamma (hypothesis 5) frequency bands. To group these features, the hypothesis that different listening tasks (LA, BA, and BAU) create a variance in the EEG features that will also be responsible for at least part of the observed differences in learning from speech in noise, was used. Variance in the EEG features between participants is likewise expected to be informative for the observed differences in learning from speech. Different techniques are available for data-driven aggregation of the broad collection of EEG features. Principal Component Analysis (PCA) of the z-score for each feature is the lowest order approach. It could be extended to higher-order statistical methods and machine learning (e.g., using deep learning auto-encoders). Because of the amount of data available and the advantage of explainable results, we decided to use PCA based on z-score normalized data. To explain the meaning of the EEG-PC scores (the representation of EEG features in the PC domain), they were compared between the listening tasks (LA, BA, and BUA) and background noises (MT, HW, and FT) using linear mixed-effect modeling (Bates et al., 2015). Assuming that the EEG PCs grasp the main variance between listening conditions observed through the different listening tasks, a supervised analysis was performed to relate them to the information acquiring and retaining z-scores (the exam results) in the lecture attended (LA) task using linear fixed and mixed-effect modeling. Also, for this predictive model, higher-order statistical approaches and machine learning techniques could have been used, yet we again opted for reducing the degrees of freedom in the model in view of the available data.

## 2. Materials and Methods

### 2.1. Participants

Twenty-three young healthy adults (mean age: 27 years, SD: 3.18, 13 females, 20 right-handed), all English speakers, participated in the experiment. Participants had normal hearing measured by pure-tone audiometry. All participants signed the informed consent and received modest financial compensation for their participation. Based on self-reports, none of them had a history of psychiatric or neurological disorders. A full battery of audiological tests was conducted including tonal audiometry, tympanometry, stapedial reflex measurement, speech in noise, and otoacoustic emissions (OAE) with contralateral suppression. No participants were excluded on the basis of this extensive testing of the auditory periphery. Our test population was young adults and therefore their hearing capabilities were fully developed (Klatte et al., 2013).

### 2.2. Tasks and Stimuli

The main stimulus was about 1 h of English lectures mixed with realistic background noise and presented through a loudspeaker while 64-channel EEG signals were recorded. Participants were instructed to pay attention to the lectures and were informed that there would be a written exam afterward. This task is hereafter referred to lecture attended (LA). The lectures were read by a male speaker and recorded in an anechoic room. To level out participants' particular interests, 13 different 5-min topics were presented over one long lecture. The lectures were related to topics for which prior knowledge is expected to be minimal in order to facilitate the focusing of attention during the presentation.

For the background noise, three 5-min realistic environmental sound fragments from continuous highway noise (HW), fluctuating traffic (FT), and multi-talker babble (MT) sounds were used. Within these fragments, a few discrete instances of very salient sounds were added. In addition, four lecture fragments were presented in silence with a low level pink noise (PK) (a.k.a $\frac{1}{f}$ noise) at a level of 35 dB(A). The signal-to-noise ratio (SNR) of lectures and background noise was set to 5*dB*, with lectures at a level of 68 dB(A) and overall background noise level at 63 dB(A). This assured that the background noise did not mask the lecture energetically. The sound levels reported here refer to the A-weighted equivalent continuous sound levels in decibels (LAeq) which were measured over about 360 s.

For the multi-talker babble sound, recordings were made at a cocktail party where about twenty people were having conversations. The recorded speech was not intelligible. A few 3-s phone ringing sounds were added to the multi-talker sound at certain times. For the highway sound, the noise of dense traffic was recorded, for which no individual car passages could be recognized. A few 5-s emergency vehicle sounds were added to the highway sound at certain times. For the fluctuating traffic sound, recordings were made at the corner of a one-way car lane with a bicycle lane next to it, close to a park. Car passages were added to the quietest periods of the fluctuating traffic noise. In addition, at certain times, a few 1-s sounds of honking car were added. The level of the salient sounds (emergency siren, phone ringing, and car's horn) was not high enough to mask the lectures energetically. The order of presentation was completely random in both lecture and background noise while assuring the two lectures in silence were not presented in succession.

The written exam was presented after the BA condition (see below), which ensured that there was a time span of 45 min between the lectures and the exam. The purpose of presenting the exam is to quantify the amount of information that participants have actually acquired and retained from the lectures. The type of questions and evaluation of the exam is explained in section 2.9. A sufficiently long time interval between the learning phase and the memory retrieval during the exam was chosen for two reasons: (1) to avoid that the last lecture would be more prominently in short term memory; (2) to avoid sequential recall as much as possible. Testing the memory and learning in a timescale of minutes and hours was discussed in Tetzlaff et al. (2012) and Kelley and Whatson (2013). For example, memory retention was tested after 30 min (Menzel et al., 2001). The choice of 45 min was a compromise between the duration of the experiment and assuring the above.

To increase the range of monitored listening conditions and to allow to implicitly calibrate for inter-person differences, the participants were exposed to two additional tasks. Firstly, as a reference for top-down attention-driven listening, 12 different 3-min fragments of background noise were presented with equivalent levels of 63 dB(A) and participants were asked to pay attention to the background noise by focusing on the number of salient events, such as phone-ringing, emergency vehicle, and honking car sounds. However, this was only to make them focus on the background sound and no questions were asked about this afterward. This task hereafter is referred to as background attended (BA). Finally, 12 different 3-min background noise fragments were presented and the participants were instructed not to pay attention to any sound, which hereafter is referred to background unattended (BUA). The BUA task is definitely different from the resting state because not paying attention to the low-level characteristics of the sounds is inevitable. Unlike the BA task, the participants during BUA were instructed not to focus on the information related to the salient events. BUA task was presented after the exam which made the participants very aware that no further attention was needed at this point, and they could relax.

**Figure 1:**
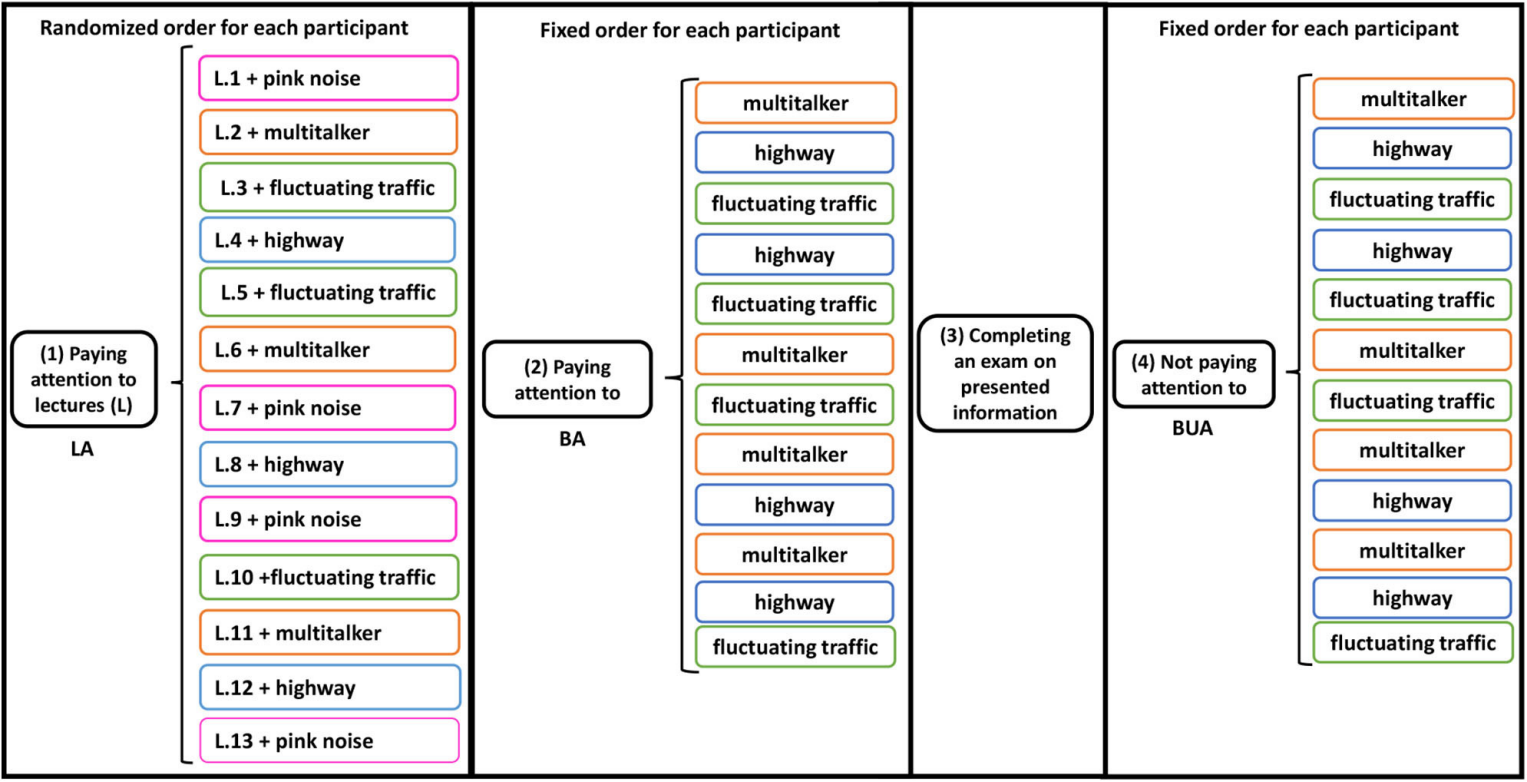
Schematic of experimental protocol and auditory stimuli presentation. Three sequential listening tasks were performed: (1) Lecture attended (LA), (2) Background attended (BA), and (3) Background unattended (BUA). In first task, in addition to multi-talker, highway and fluctuating traffic sounds as the background noises, the lectures were also presented in pink noise and without any background noise. After second task, a written exam was asked to complete about vocal information in the first task. Equivalent levels of background noise and lecture were ~63 and 68 dB(A), respectively. The lectures were are shown by L.*i, i* = 1, …, 13, and the noises are distinguished by different colors in the figure.

By listening task (or simply task) along with this paper, we mean the tasks that the participant had to perform during the experiment, i.e., LA, BA, and BUA. By listening condition (or simply condition), we mean the conditions that the subjects were flooded with the listening tasks and the stimuli. In total, all subjects were exposed to ten different listening conditions depending on the task and noise: LA-PK, LA-MT, LA-HW, LA-FT, BA-MT, BA-HW, BA-FT, BUA-MT, BUA-HW, BUA-FT. For instance, LA-MT refers to the condition that the task is LA and the background noise is MT. The experimental protocol is schematized in Figure 1.

**Figure 2:**
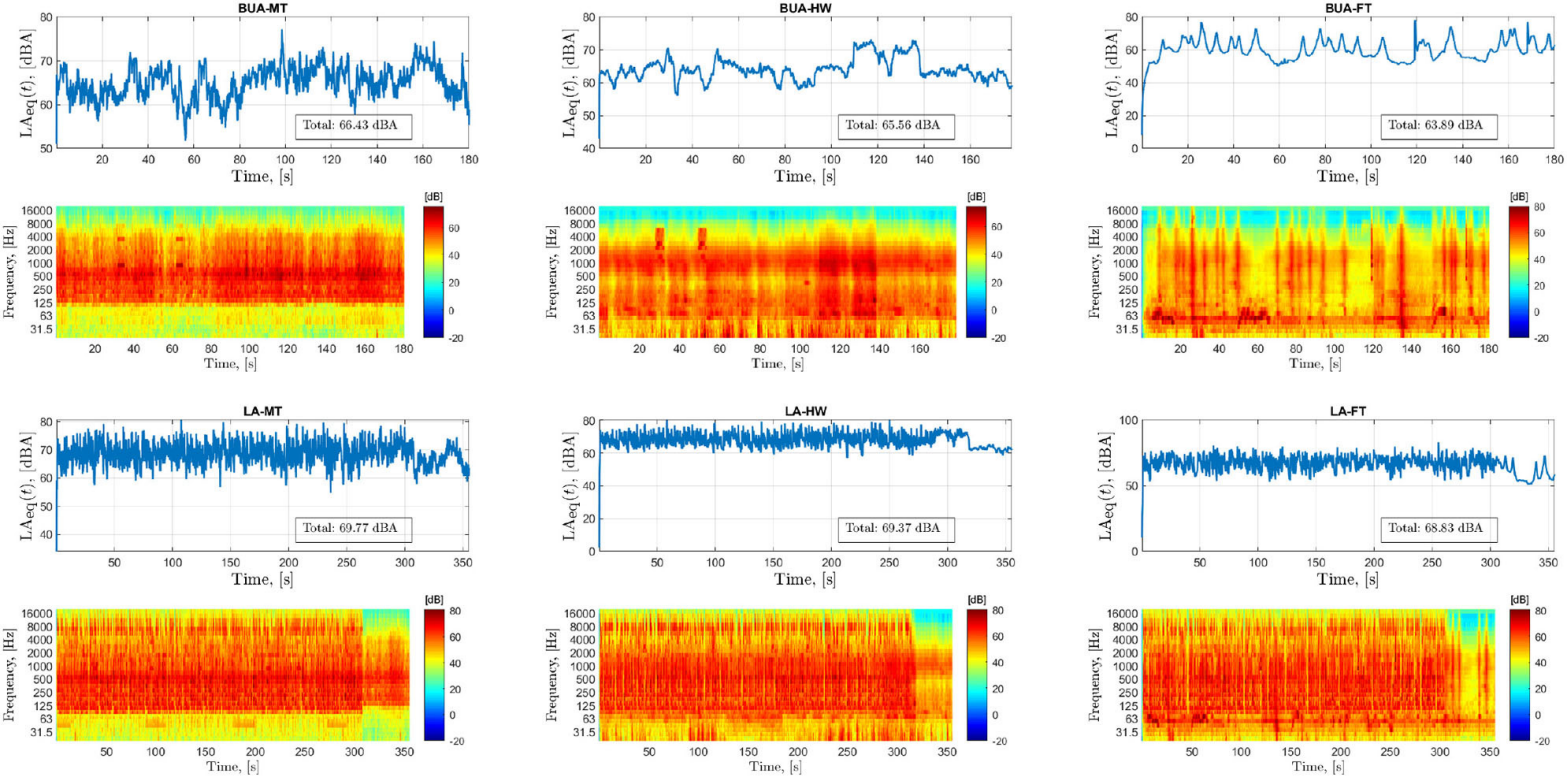
Acoustic characteristics of stimuli presented in different listening conditions. Each panel corresponds to one of the sound fragments presented during the conditions labeled at the top of each panel. The line plots show the sound level fluctuation with time (A-weighted, equivalent continuous sound level in decibels, LAeq). The averaged LAeqs over the whole duration of the fragment have been annotated in the line plots. The heatmaps show the standard spectrogram of the sound fragments (with time resolution of 0.01 s and one-third octave frequency bands). LA, lecture attended; BUA, background unattended tasks; MT, multi-talker; HW, highway; FT, fluctuating traffic background noises.

Figure 2 depicts the sound level fluctuations as a function of time (line plots) and standard spectrograms (heatmaps) for one of the sound fragments presented during the LA and BUA listening tasks. From Figure 2, the FT background noise stands out in terms of sound level fluctuation. For the HW background noise, the sound level is quite stable. Finally, the MT background noise exhibits somewhat more fluctuations in the sound level than the HW noise, but the differences between the loudest and the quietest sounds levels are higher in the FT.

Note that the background sounds used in the LA and BUA tasks were the same (except the time duration). Furthermore, the type and order of background sounds presented in BA and BUA were identical for all participants. The only difference between the stimuli presented during BA and BUA is that additional salient sounds were added in the last three fragments of BA due to the increased chance of focusing on the background noise sound in the BA task.

### 2.3. EEG Recording

EEG signals during the different listening conditions were acquired continuously using a BioSemi System (Amsterdam, NL) from 64 active electrodes placed according to the standard 10−20 layout (Oostenveld and Praamstra, 2001) at a sampling frequency of 2, 048 Hz. Subjects were asked to keep their eyes open and focus on a dot located in the center of the monitor to minimize eye movement. Signals from seven external electrodes were also recorded which were applied to the nose, neck, two left & right mastoids (M1 and M2), left (HEOGL) & right (HEOGR) outer canthi, and below the left eye (VEOGD). In addition, two external channels were used for capturing the sound signals (SoundL and SoundR) together with EEG signals.

### 2.4. EEG Pre-processing

The EEG data were offline re-referenced to the nose electrode (channel 65th) and re-sampled to 512 Hz using an anti-aliasing finite impulse response (FIR) low-pass filter. The EEG data were then filtered using an FIR bandpass filter (Hamming windowed sinc) of order 3,380 from 0.5 to 134 Hz to remove extremely slow drifts and sharp oscillations.

EEG signals were cleaned up in two steps. At first, non-repeating big artifacts were removed based on visual inspection. In a second step, infomax independent components analysis (ICA) (Bell and Sejnowski, 1995) with EEGLAB version 13.1.1b (Delorme and Makeig, 2004) using default settings was applied to identify and remove eye blink and movement artifacts. To identify the ICA components related to eye artifacts, some rules of thumbs were applied: (1) no more than three ICA components were removed; (2) both temporal and spatial plots should confirm the diagnosis of eye artifact, meaning frontally located components and a typical blink or nystagmus pattern; (3) in case of doubt, the temporal pattern of the supposed ICA component was compared with the temporal pattern of the Electrooculography (EOG) channels to make sure that the incidence of potential eye artifacts coincide; (4) only eye artifacts were removed.

Since playing audio files typically has a latency of a few milliseconds, the sound was recorded together with the EEG on a free channel which could be used to synchronize with the presented audio signal. For this purpose, at first, the presented audio files were re-sampled to 512 Hz (using an anti-aliasing FIR low-pass filter) and then the cross-correlation between re-sampled audio signals and recorded sound signals together with the EEG was calculated. The lag corresponding to maximum cross-correlation is the delay in audio files with respect to EEG measurement. To compensate for this delay, all 64-channel EEG signals were shifted with estimated delays. For the analysis in this manuscript, this synchronization is less important.

Finally, the power spectrum plots of all EEG channels were visually inspected, and the fragments whose all channels were extremely noisy were excluded. In addition, using the power spectrum and a combination of visual inspection and automatic method (median-based criteria), the channels that were extremely noisy were excluded.

### 2.5. EEG Signal Processing

First, the continuous EEG signals were split into separate fragments corresponding to the 3 or 5 min exposures, based on sound signal recorded as extra EEG channel. Each EEG fragment was then analyzed per channel. Three types of EEG feature were estimated: (1) low-frequency-based features, such as absolute and relative powers, bandwidth, central frequency and spectral edge frequency for delta and theta activities, (2) alpha-band based features, such as alpha peak frequency and power, individual alpha frequency, absolute and relative powers, and alpha-band scaling exponent value as a dynamic measure to quantify LRTC, and (3) high-frequency-based features, such as bandwidth, central frequency, and spectral edge frequency for the beta and gamma signals. Moreover, wide-band absolute power, theta/alpha ratio power, and absolute power for lower and upper alpha were estimated. The subsequent sections describe how a broad range of EEG features was estimated.

#### 2.5.1. Power Spectra-Based Features

To estimate the power spectrum density, Welch algorithm was applied. We used 1 s hamming window with 0.5 s overlap, 2^14^ frequency bins, and frequency sampling of 512 Hz. The power spectrum density, **p**, is estimated for the frequency range **f** ← [0 − 256 *Hz*] with frequency resolution of $\frac{2^{6}}{2^{14}}$ Hz. In addition to six fixed frequency bands including δ (1 − 4 Hz), θ (4 − 8 Hz), α (8 − 13 and 7 − 13 Hz), β (13 − 30 Hz), and γ (30 − 45 Hz), the lower (8 − 10 Hz), upper (10 − 13 Hz) α band, and the wide-band (1 − 45 Hz) were separately analyzed.

Absolute and relative powers (AP and RP) were calculated from the 64 scalp locations in the mentioned frequency bands. Relative power was computed as the ratio of power in a given band to sum of power from 1 to 45 Hz. Moreover, the $\frac{\theta }{\alpha }$ power ratio (RPTA) was computed. For the frequency band 1 to 45 Hz, only the absolute power was computed. In addition to these power-based features, the following frequency-based features (Szeto, 1990; Drummond et al., 1991; Estrada et al., 2004; Vural and Yildiz, 2010) were computed for the different frequency bands using the definitions in Vural and Yildiz (2010): (1) central frequency, (2) bandwidth, and (3) spectral edge frequency 95%. The central frequency (CF) is defined as the center of gravity for frequency between the lower and upper cutoff frequencies of the power spectrum. The bandwidth (B) quantifies the width of the power spectrum over a specific central frequency. The spectral edge (SE) frequency 95% is defined the frequency below which 95% of the total power (in a specific frequency band) are located (Szeto, 1990).

#### 2.5.2. Alpha Peak Frequency Based on Root-MUSIC

To estimate the alpha peak frequency and power, we used the root-multiple signal classification (root-MUSIC) algorithm (Barabell, 1983). The root-MUSIC as a subspace-based method estimates the frequency content of a signal using an eigenspace method. The root-MUSIC algorithm has been described in recent work from our group (Eqlimi et al., 2019). In this paper, the preprocessed EEG signals were band-pass filtered at 7 − 13 and 8 − 13 Hz (using Butterworth band-pass filter of order 2) for two reasons: (1) there is no consensus on the alpha range (like other frequency bands) and both lower cutoff frequencies (7 and 8 Hz) have been used in literature (Freeman and Quiroga, 2012; Clayton et al., 2015); (2) it has been shown that there is a 2.8 Hz between-subject variability (mean = 10.3 Hz) for the alpha peak frequency (Haegens et al., 2014). The root-MUSIC algorithm was performed on each filtered EEG channel with *P* = 2 as the dimension of the signal subspace. The maximum powers in μV^2^ and corresponding frequency in Hz were found. MP2713 and MP2813 terms (which are used in the following sections) stand for MUSIC-based alpha peak power which are estimated in alpha frequency ranges of 7 − 13 and 8 − 13 Hz, respectively with *P* = 2 components. The corresponding alpha peak frequencies are denoted by MF2713 and MF2813.

#### 2.5.3. Individual Alpha Frequency Based on Fitting Process

Individual alpha frequency (IAF) could also be estimated based on the Gaussian fit approach (Nikulin and Brismar, 2006; Van Albada and Robinson, 2013; Haegens et al., 2014). We employed the algorithm which has been suggested in Neurophysiological Biomarker Toolbox (NBT) version 0.5.5 (Hardstone et al., 2012) to quantify IAF. Firstly, PSD (**p**) and its corresponding frequencies (**f**) of each EEG signal with a 0.1 Hz resolution were estimated. The peak amplitudes and locations of **p** in the range of 8–13 Hz were found (using Matlab function “findpeaks”). A polynomial (*y*_0_ = *p*_1_*x* + *p*_2_) function was fitted to ln(**p**) for considering a $\frac{1}{f}$ baseline. Then, $\text{z}\leftarrow e^{p_{2}}+{\text{f}}^{p_{1}}$ and **s** ← **p** − **z** were calculated to remove the $\frac{1}{f}$ component of the spectrum (Nikulin and Brismar, 2006).

A Gaussian function, $y_{1}=a_{1}e^{-{\left(\frac{x-b_{1}}{c_{1}}\right)}^{2}}$ was fitted to the detrended power spectrum, **s**, to consider one peak. 95% prediction bounds, i.e., confidence interval, [*cl*_*l*_, *cl*_*u*_] for *a*_1_ and *b*_1_ were calculated. If *a*_1_ + *y*_0_(*b*_1_) > *cl*_*u*_, then *f*_α_ ← *b*_1_ and *p*_α_ ← *a*_1_. To determine IAF, center of gravity within the alpha band could be estimated. At first, the individual frequency interval, namely [*f*_1_, *f*_2_] ← [TF, |5 − (*f*_α_ − 1)| + *f*_α_] is calculated. TF stands for transition frequency and defined as the EEG frequency lower than the alpha peak frequency showing the minimum power (Klimesch, 1999). Then, the center of gravity was calculated using $\text{IAF}\leftarrow \frac{\overset{f_{2}}{\underset{k=f_{1}}{\sum }}\text{f}\left(k\right)\text{p}\left(k\right)}{\overset{f_{2}}{\underset{k=f_{1}}{\sum }}\text{p}\left(k\right)}$. Finally, *f*_2_ was updated by *f*_2_ = |5 − (IAF − 1)| + IAF and IAF was re-calculated based on same definition. Compared to root-MUSIC based alpha peak frequency (MF2813), the IAF is expected to be less sensitive to bandwidth around the observed frequency, yet both parameters are highly correlated.

#### 2.5.4. Long-Range Temporal Correlations of Alpha Activity

Processes that do not have a characteristic scale (i.e., scale-free processes) cannot be described completely in terms of spectral concepts (e.g., peak frequency). There is convincing evidence that EEG time series exhibit scale-free dynamics (He, 2014). One of the successful methods to analyze these scale-free signals is long-range temporal correlations (LRTC). LRTC has been developed to quantify how much future dynamics of a signal are influenced by past temporal events (Linkenkaer-Hansen et al., 2007).

In fractal geometry, LRTC could be interpreted by a self-similarity behavior, which suggests the signal dynamics are similar in different time scales. One of the most common techniques to quantify LRTC is detrended fluctuation analysis (DFA) (Peng et al., 1994). The presence of a trend in the signal can cause an overestimation of LRTC, hence DFA tries to eliminate the trend. Indeed, DFA is employed to quantify how slowly the autocorrelations of signals decay in power law, which is called the scaling exponent value, *a*. The power or scaling law states that a relative change in one quantity results in a proportional relative change in another, namely one quantity varies as a power of another. Distributions of the form *p*(*x*) = *Cx*^−*a*^ are said to follow a power law. The constant *a* is called the exponent of the power or scaling exponent value (SEV) (Newman, 2005). If 0.5 < *a* < 1, the signal likely exhibits strong LRTC (Hardstone et al., 2012).

We employed the DFA algorithm to quantify LRTC for each EEG channel signal in the alpha band using the NBT version 0.5.5 as suggested in Hardstone et al. (2012). First, the EEG signals were band-pass filtered from 8 to 13 Hz (alpha range used in Hardstone et al., 2012) using the Hamming windowed FIR filter of order 0.25 s (2 cycles of the lowest frequency, 8 Hz). Second, the amplitude envelope of the band-pass filtered signal was estimated based on the Hilbert transform. Third, the cumulative sum of the amplitude envelope was calculated as follows:

(1) $$\text{c}(t)=\sum_{k=1}^{t}\text{e}(k)-\bar{\text{e}},$$

where **e**(*k*) is the amplitude envelope at time instant *k*, $\bar{\text{e}}$ is mean of the amplitude envelope, and **c**(*t*) is the cumulative sum of amplitude envelope at time instant *t* (a.k.a signal profile). We defined a set of window size, **s** = {*s*_1_, …*s*_*N*_}, which are equally spaced on a logarithmic scale in a predefined calculation range. The cumulative sum of amplitude envelope (**c**(*t*)) was then split into a set of *n* separated time windows of length ∀ *l* ∈ **s**, which have 50% overlap. For each time window, the linear trend was removed using a least squares method and obtained the detrended version. After calculating the standard deviation of the detrended time windows, the fluctuation function as the mean standard deviation of all windows was computed as follows:

(2) $$\bar{\text{f}}(l)=\frac{1}{n}\sum_{i=1}^{n}\sigma_{{\hat{\text{w}}}_{i}^{l}},\;l\in \text{s},$$

where $\sigma_{{\hat{\text{w}}}_{i}^{l}}$ is the standard deviation of *ith* time window of length *l* ∈ **s**, *n* is the number of time windows. Finally, we plotted the fluctuation function, $\bar{\text{f}}\left(l\right)$, along *l* on logarithmic axes. The slope of the trend line was computed in a predefined fitting interval using the linear regression as a measure for LRTC which is called scaling exponent value (SEV). Two different calculation ranges of 2.5–180 s and 0.1–180 s were evaluated (SEV1 and SEV2, respectively). A fitting interval of 5–18 s was considered such that the filter effect is negligible (Hardstone et al., 2012). The signal length in the LA task was about 360 s, whereas the signal length in the BA and BUA tasks was about 180 s. To minimize the effect of signal length, 180 s was selected as the upper bound of calculation range for the three listening tasks.

### 2.6. Unsupervised Analysis Using Principal Component Analysis

Let **X** ∈ ℝ^*n*×*p*^ contains *n* observations of *p* EEG features, where could be obtained by concatenating the EEG features per participant, channel, stimulus, and condition in rows. In order to emphasize variation and identify strong patterns in EEG features, a principal component analysis (PCA) was applied on **X** which is a broad dataset including explicit listening conditions and persons. All power-based EEG features (i.e., absolute and peak powers) were mapped to logarithmic scale (log-transforming) before applying PCA. Since the EEG features do not have the same scales, the data was normalized using z-score transformation such that each column of **X** re-centered to have zero mean and scaled to have a unit standard deviation.

PCA seeks a linear combination of features such that the maximum variance is extracted from the feature. One of the methods of performing PCA is the singular value decomposition (SVD) method. The SVD decomposes **X** into three matrices, i.e., **X** = **USV**^T^. The PCA results are expressed by two matrices: (1) the PC loadings (coefficients), **V** ∈ ℝ^*p*×*p*^, can be understood as the weights for each original variable when calculating the principal component; (2) the PC scores (PCSs), **Us**^T^ ∈ ℝ^*n*×*p*^ referring to the representation of **X** in the PC space, where **s** ∈ ℝ^*p*×1^ is the vector containing the main diagonal elements of **S** (i.e., the singular values). In other words, each observation in the original space may be projected onto a given PC in order to get a coordinate value along the PC-line. This new coordinate value is known as the PC score. The PC scores are the representation of **X** in the PC space. In fact, the PC scores can be calculated with **X**/**V**^T^.

### 2.7. Grouping the Channels in Subregions

The 64 EEG channels were labeled with six fixed subregions: frontal, central, left and right temporal, parietal, and occipital. This division, while allowing four main lobes of cerebrum (Graimann et al., 2010), also considers the central region and left & right hemispheres for the temporal lobe. A similar grouping of channels has been used in previous studies, e.g., for the short-term memory task (Schack et al., 2002). Although subsequent analyses are presented in section 2.8 was performed per channel, the subregion was used a categorical fixed factor in the mixed modeling of EEG-PC scores. However, EEG-PC scores averaged across subregions were used to model the exam result (section 2.9).

### 2.8. Statistical Analysis of EEG-PC Scores

Linear mixed-effect modeling (LMEM) was used to model the EEG-PC scores as a linear combination of the predictors using the LME4 package (Bates et al., 2015) of the statistical software R (R Core Team, 2019) to explain to origin of EEG-PC scores. The LMEM extends the general linear models (GLMs) to allow both fixed and random effects. A fixed effect is a constant variable across individuals while a random effect varies across individuals. Different LMEMs have been built separately for the nine response variables (EEG-PC scores) as a function of the fixed and mixed (random) effects of interest. Since the person-dependent effects may not be captured in the response variables, the participant variable has been considered as a random effect in all the LMEMs.

On the one hand, the EEG-PC scores were modeled as a function of task type and channel subregion for each specific background noise type based on formula (3), which is hereafter referred to within-background modeling:

(3) $$\begin{array}{l} {\text{LMEM}}_{\text{within-background}}\leftarrow \text{PC}{\text{S}}_{i}^{j}\sim (1|\text{participant})+1\\ +\;\text{task}+\text{subregion}. \end{array}$$

In formula (3), ${\text{PCS}}_{i}^{j}\in {\mathbb{R}}^{n_{j}\times 1}$ is a vector including *ith* EEG-PC scores for *jth* background noise and all 64 EEG channels, where *i* = {1, …, 9}, *j* = {1, …, 4} and *n*_*j*_ is the number of observations belonging to all listening tasks in jth background noise. The symbol “~” implies that left term is modeled as a function of right terms. The fixed effects include **task** and **subregion**. The constant and random terms are expressed in **1** and (**1**|**participant**), respectively, where **participant** is a categorical variable that has 23 possible outcomes. The term **task** includes the listening task types and has three possible values: lecture attended (LA), background attended (BA), and background unattended (BUA). The last term, **subregion**, is another categorical variable and has six possible outcomes: frontal, parietal, occipital, central, left, and right temporal. Since for each type of background noise, one model is separately defined, no interaction between task and background noise type can be considered.

On the other hand, the EEG-PC scores were modeled as a function of background noise type and channel subregions for each specific listening task based on formula (4), which hereafter referred to within-task modeling:

(4) $$\begin{array}{l} {\text{LMEM}}_{\text{within-task}}\leftarrow \text{PC}{\text{S}}_{i}^{k}\sim (1|\text{participant})+1\\ +\;\text{background}+\text{subregion}. \end{array}$$

In formula (4), ${\text{PCS}}_{i}^{k}\in {\mathbb{R}}^{n_{k}\times 1}$ is a vector including *i*th EEG-PC scores for *k*th listening task noise and all 64 EEG channels, where *i* = {1, …, 9}, *k* = {1, 2, 3}, and *n*_*k*_ is the number of observations belonging to all background noises tasks for *k*th listening task. The term **background** includes the background noise types and takes four possible values: pink (PK), multi-talker (MT), highway (HW), and fluctuating traffic (FT).

After estimating the coefficients (intercept and slope) for each fitted model, general linear hypotheses and Tukey *post-hoc* multiple comparisons were then performed to test for the significance of EEG-PC scores changes across the task and background types. For example, we may consider the six pairwise comparisons between the background noises for the fitted model of the first EEG-PC score in the LA task. The question is which specific background's means (compared with each other) are different. A pairwise Tukey's test examines more than one pair of means the same time and corrects for family-wise error rate.

### 2.9. Statistical Analysis of Exam Results

As mentioned in the section 2.2, we performed a written exam to check the participant's learning during the lecture attended (LA) task. The exam was carried out after all lectures and the attentive listening to background sounds (see Figure 1). Open and closed questions were asked per topic. Open questions were either factual or insight questions. Closed questions consisted of sentences that had to be completed with a specific word or number. The questions were carefully designed so that the answers could be found well-spread over the whole lecture. Over the different topics, the order of question types was randomized. For the open questions, the answers could always be found in three or four connected sentences.

The total number of keywords vary per topic. This was deliberately done to capture as closely as possible anything the participants might have recalled, which is important for the EEG analyses (distinguishing between attention and no attention with remembered keywords as ground truth). The topics of the lectures were chosen to avoid prior knowledge by the participants, yet some topics may be more difficult to grasp than others for the average participant. Moreover, there could be small differences in difficulty between the questions. Prior knowledge and logical reasoning of listeners about the answers are not reflected in listening conditions (background sound) nor in the EEG during listening. Therefore, the number of correctly retained keywords was normalized per participant, background noise and topic and the exam z-scores were calculated as follows:

(5) $$\text{Exam z-score}=\frac{\#\text{Correctly Retained Keywords}-\mu_{\text{pink}}}{\sigma_{\text{pink}}},$$

where μ_pink_ and σ_pink_ are the mean and standard deviation of the number of correctly retained keywords across all subjects for each topic presented in pink noise (LA in silence), respectively. This a fair reference, as all topics are sufficiently represented in silence.

To validate the predictability of exam results by a linear combination of the EEG-PC scores, we used the linear fixed and mixed-effect modeling as explained in the previous section. In fact, the response variable here is the exam z-scores and the EEG-PC scores are considered as the predictors. Moreover, to show that the EEG contains more information than the listening condition, the exam results was also modeled as a function of background noise type and performance was compared to the models based on EEG.

Person-dependent differences in the exam results may include the following: mental state, traits, physiological differences, prior knowledge, etc. Some of these differences may reflect in EEG, others may not. Hence it is useful to use both linear fixed and mixed-effect modeling. Linear fixed-effect model regresses the exam results as a function of desired fixed factors without considering participant as a random factor, whereas linear mixed-effect model includes participant as a random effect to capture between-subject variability. The latter implies that a fixed offset in exam results per participant is included in the model. Linear fixed-effect models (LFEM) are expressed in the following formulas:

(6) $${\text{LFEM}}_{\text{constant}}\leftarrow \text{exam}\;\text{z-scores}\sim 1,$$

(7) $${\text{LFEM}}_{\text{background}}\leftarrow \text{exam}\;\text{z-scores}\sim 1+\text{background}\;\text{type},$$

(8) $${\text{LFEM}}_{\text{EEG}}\leftarrow \text{exam}\;\text{z-scores}\sim 1+\sum_{i=1}^{i=9}\sum_{j=1}^{j=6}\text{PC}{\text{S}}_{ij}^{\text{Avg}},$$

where exam z-scores (as the response variable) were defined by a vector including all exam z-scores computed by Equation (5). ${\text{PCS}}_{ij}^{\text{Avg}}$ includes *i*th EEG-PC scores for *j*th channel subregion for lecture attended task in all background noises, which were obtained by averaging the PC scores across the channels corresponding to the given subregion (see the section 2.7). The background type term is a categorical variable that has four possible outcomes: pink, multi-talker, highway, and fluctuating traffic.

Similarly, linear mixed-effect models (LMEM) could be expressed in

(9) $${\text{LMEM}}_{\text{constant}}\leftarrow \text{exam}\;\text{z-scores}\sim (1|\text{participant})+1,$$

(10) $$\begin{array}{l} {\text{LMEM}}_{\text{background}}\leftarrow \text{exam}\;\text{z-scores}\sim (1|\text{participant})+1\\ +\text{background}\;\text{type}, \end{array}$$

(11) $$\begin{array}{l} {\text{LMEM}}_{\text{EEG}}\leftarrow \text{exam}\;\text{z-scores}\sim (1|\text{participant})+1\\ +\sum_{i=1}^{i=9}\sum_{j=1}^{j=6}\text{PC}{\text{S}}_{ij}^{Avg}, \end{array}$$

where exam z-scores and ${\text{PCS}}_{ij}^{\text{Avg}}$ are defined same as the linear fixed effect models. The constant and random terms are shown by **1** and (**1**|**participant**), respectively.

Since 54 EEG-PC scores (the 9 components for each of the 6 subregions) are available to regress exam z-scores, a stepwise regression method can be used to choose the most contributing predictive variables. The backward-elimination approach was applied on both full models (LFEM_EEG_ and LMEM_EEG_). To this end, we used “step” function in “STATS” v3.6.2 package of the statistical software R (R Core Team, 2019). This function starts from 54 candidate variables, tests the effect of the deletion of each variable using the Akaike information criterion (AIC) (Akaike, 1974), deletes the variable whose loss gives the least statistically insignificant deterioration of the model fit, and repeats this process until no further variables can be deleted without a statistically significant loss of fit.

## 3. Results

The results consist of two parts: (1) unsupervised analysis of the EEG features observed under different listening conditions (sections 3.1–3.3) and (2) supervised analysis to predict the exam results in lecture attended task (section 3.4). Section 3.1 presents the loading of principal components (PC) on underlying features; section 3.2 demonstrates the scalp topographies of the PC scores; and section 3.3 explains the relationship between PC scores, listening conditions and backgrounds. In the last section, a supervised training of models was used to investigate the predictability of acquiring and retaining performance scores (exam results) by EEG-PC scores.

### 3.1. Principal Component Analysis

**Figure 3:**
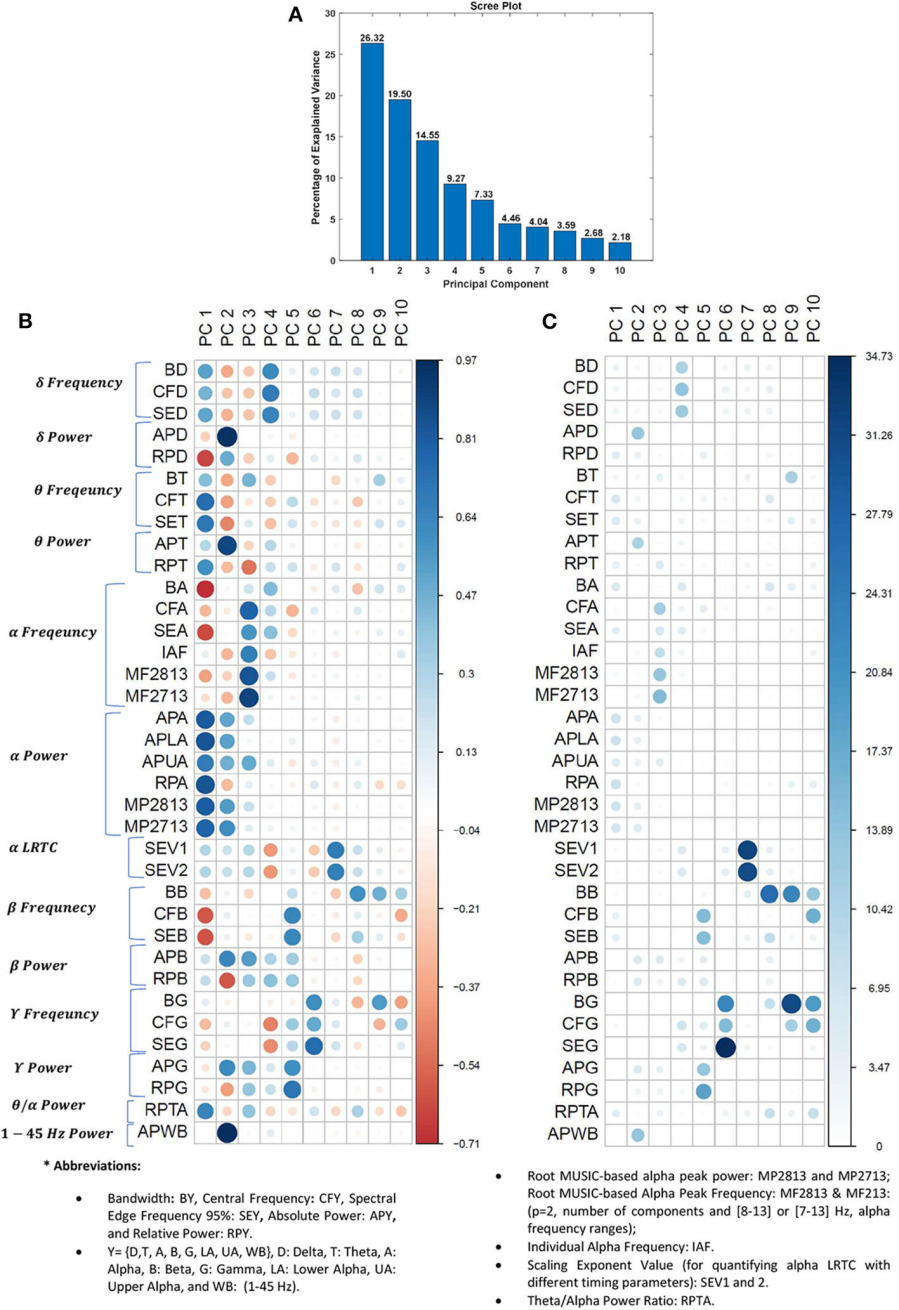
PCA on EEG features. **(A)** Scree plot displays the percentage of explained variance in a downward curve, ordering the eigenvalues from largest to smallest. **(B)** The coordinates of EEG features in PC domain in the rows. The positive and negative correlation values between features and PCs are visualized by cool and warm colors, respectively. **(C)** The contribution of EEG feature to the PCs in percentage, i.e., the squared coordinates were normalized to total sum of squared coordinates on the PCs. The larger and darker circles indicate the EEG features contributes more to the given component. The difference between **(B,C)** is that the **(B)** shows the correlation between features and PCs, while **(C)** shows the representation quality of features on the PCs (i.e., normalized squared correlation values in percentage).

The explained variances by the ten most important principal components in percent are displayed in Figure 3A. Together these ten components explain about 94% of the variability in the dataset. The coordinates of individual EEG feature in principal component (PC) domain are visualized in Figure 3B. The correlation between a feature (variable) and a PC is used as the coordinates of the variable on the PC. The size and darkness of circles in Figure 3B is proportional to the correlation value between an EEG-feature and a given PC. The positive and negative correlation values are visualized by cool and warm colors, respectively.

Figure 3C visualizes the contribution of EEG features to the PCs in percentage. The contribution of *ith* EEG feature to *jth* PC is expressed in $\frac{{\left(y_{ij}\right)}^{2}}{\overset{n}{\underset{j=1}{\sum }}{\left(y_{ij}\right)}^{2}}\times 100$, where *y*_*ij*_ is the coordinate of *ith* EEG feature on *jth* PC and *n* = 10 is the number of PCs. In fact, in Figure 3C, the squared coordinates were normalized to total sum of squared coordinates on the PCs. The squared coordinates can be a quantity to measure the quality of representation of the features on PC domain.

As can be seen from Figures 3B,C, the different features contribute to each component. Accurate grouping of these PCs is not possible due to presence of different positively and negatively correlating features with the PC scores (Figure 3B). It is worth noting that normalized version of squared coordinates (Figure 3C) shows that the last five PCs have more specific loading (representation quality) than those of first five PCs. Specifically, the long-range temporal correlations of alpha band and frequency information of gamma and beta bands are most contributing features to represent PC domain.

### 3.2. Scalp Topographic Maps

**Figure 4:**
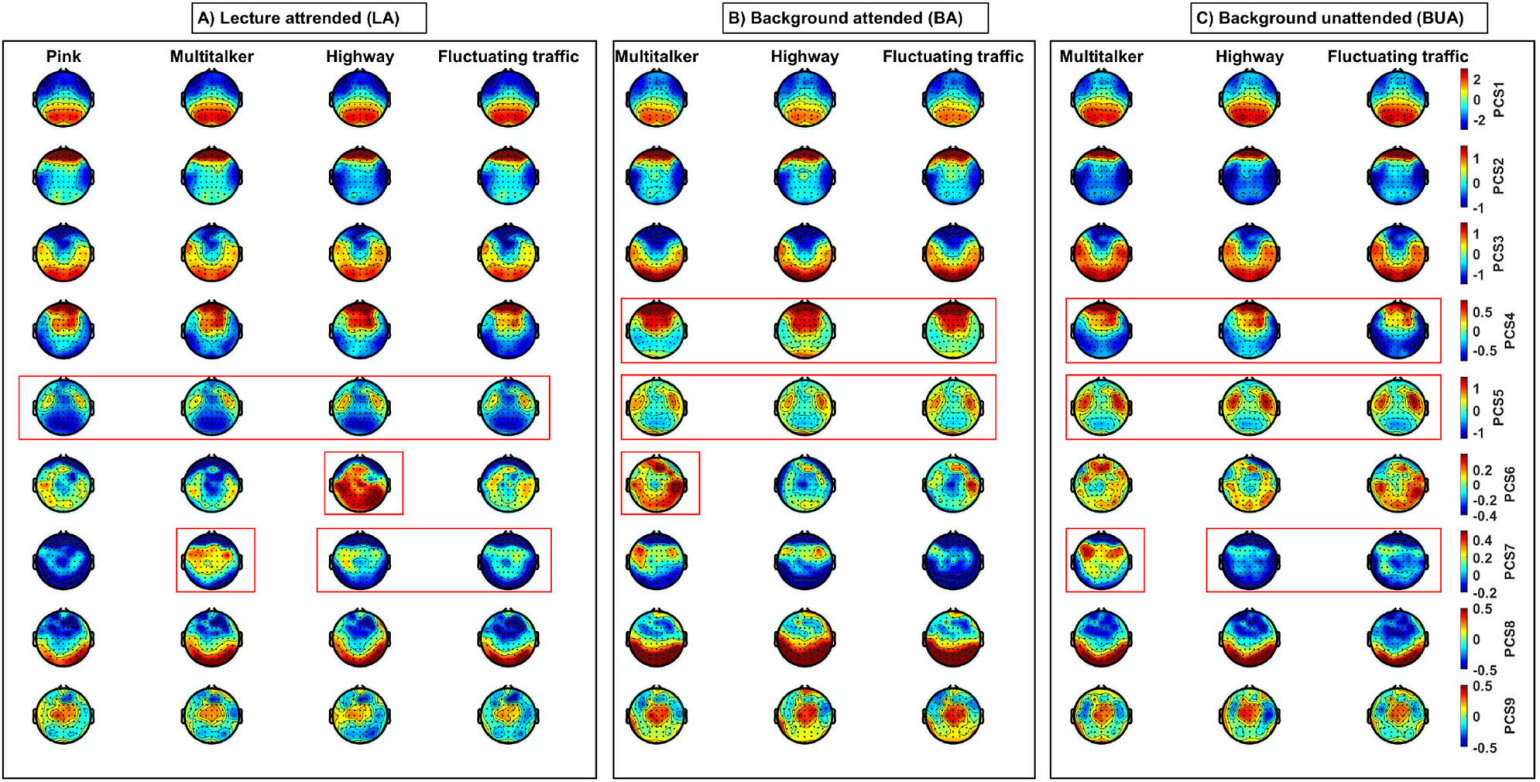
Topographic maps of nine first EEG-PC scores (**PCS**i, i = 1, …, 9) of EEG-features (in rows) for three listening tasks (in three panels): **(A)** lecture attended (LA); **(B)** background attended (BA); and **(C)** background unattended (BUA) in different noises. Heads are in vertex view, nose is above, and left ear is on the left side. Each topographic map has been obtained by averaging EEG-PC scores across participants and fragments per listening condition and EEG channel. Each column belongs to one specific listening condition. The type of background noises is shown above the corresponding columns. The warm and cool color-coded areas represent the positively and negatively correlated cortical areas with the extracted components, respectively. Red frames show some spatial and activation differences suggesting the EEG-PC scores might contribute to statistical significance in the discrimination between the three listening tasks and the three background noises. Specifically, (i) PCS4 is higher in BA compared to BUA, (ii) PCS5 is the maximal and minimal in BUA and LA, respectively, (iii) PCS 6 is the maximal for highway in LA and multi-talker in BA, and (vi) PCS7 is the maximal for multi-talker in LA and BUA.

For visualization across the scalp, 2D topographic maps of the component scores are shown in Figure 4. The topographies of the nine first PC scores (**PCS**_*i*_, *i* = 1, …, 9) were obtained by averaging across all subjects and the specific fragments for each listening conditions. In fact, for *c*th EEG channel, *j*th listening task, and *k*th noise, the average value of *i*th PC scores was calculated using ${\bar{\text{PCS}}}_{i}^{jk}\left(c\right)=\overset{N}{\underset{p=1}{\sum }}\overset{l}{\underset{f=1}{\sum }}{\text{S}}_{p}^{f}\left(c\right)$, where $\text{S}={\text{PCS}}_{i}^{jk}$, *N* = 23, and *l* are the number of participants and stimulus fragments, respectively.

Note that here we do not aim at reporting the statistical differences between the listening conditions in terms the PC scores. However, some spatial and activation differences can be observed between different listening conditions (shown by red frames in Figure 4) suggesting the EEG-PC scores might contribute to statistical significance (refer to section 3.3) in the discrimination between the three listening tasks and the three background noises. Each component is a linear combination of different positively and negatively correlated features with the components (refer to Figure 3). Therefore, in Figure 4, both of the warm and cool color coded areas are important, which represent the positively and negatively correlated cortical areas with the extracted components, respectively.

The qualitative differences of some components between different conditions have been shown by red frames in Figure 4. Specifically, the PCS 5 is the lowest in the LA compared to other tasks, the PCS 6 is the highest in the highway during the LA, and the PCS 7 is the highest in the multi-taker for the three tasks. In addition, these topographies indicate that different PCs contribute to different cortical areas. For example, the third PC score is positively dominant over temporal and occipital regions.

### 3.3. Explainable Origin of EEG-PC Scores

The unsupervised extraction of PCs from our dataset implicitly attempts to discriminate between participant, listening task (LA, BA, BUA), and background (MT, HW, FT). One way to analyse the origin of a PC is to construct a regression model for its score based on the above-mentioned factors as explained in section 2.8.

A constant mixed-effect model for predicting EEG-PC scores is expressed in [**PCS**_*i*_ ~ (**1**|**participant**)], where **PCS**_*i*_ is *i*th PC score for all listening conditions and channel subregions. If channel subregion is added as a fixed factor to the constant model, the new model could better predict all PC scores (*p* < 10^−15^) compared to the constant model. By adding listening task type to the current model, all PC scores except the sixth PC score are better predicted (*p* < 10^−8^). Background noise type as an additional fixed effect could improve the current model for all PC scores except the ninth PC score (*p* < 0.05). By adding interaction between background noise and task types, the improvement of current model is significant for all PC scores (*p* < 10^−4^) except the ninth PC score. Since the interaction between task and background noise type significantly improves modeling EEG-PC scores, its effect was separately investigated using two distinct models, within-background and within-task modeling based on formulas (3) and (4), respectively.

Tukey *post-hoc* multiple comparison for within-background and within-task models were reported in Tables 1, 2, respectively (refer to section 2.8). Each test in the sub-matrices was run independently. For example, for a particular background type and PC score, the listening conditions are compared. In each 4 × 4 and 3 × 3 sub-matrices, upper triangular elements denote *p*-values of significant differences for corresponding comparisons, lower triangular elements denote which noise or task results in higher values of the given EEG-PC score and main diagonal elements denote which background noise or task results in the maximum/minimum values of the given EEG-PC score. For example, in Table 2, MT **PCS**_1_ is significantly higher than that of PK during LA because *e*_1, 4_ and *e*_4, 1_ elements of matrix corresponding to LA and **PCS**_1_ are < 0.001 and an arrow directed toward MT, respectively. Note *e*_*i, j*_ represents the element at the *i*th row and *j*th column of the sub-matrix.

**Table 1:**
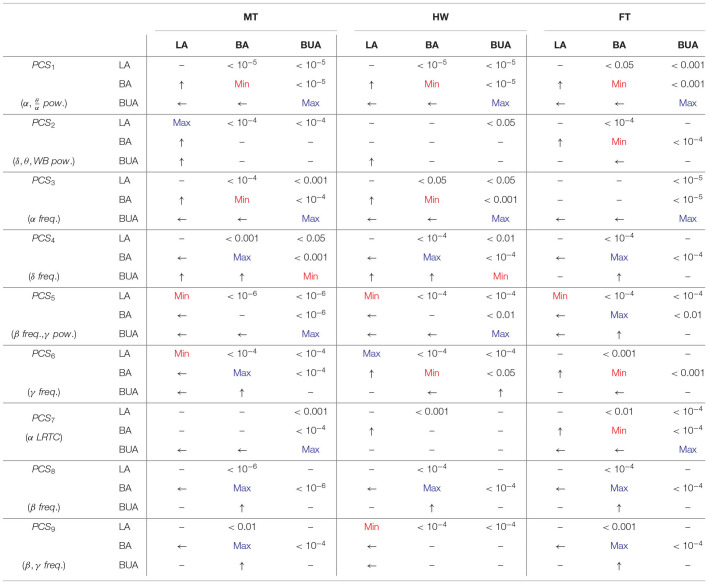
Tukey *post-hoc* multiple comparison testing for within-background model.

*Tukey variable is the task type (LA, BA, and BUA) and all possible pairs of means in each subtable are compared. Significant p-values are reported in upper triangular. Main diagonal denotes which task is the maximum (Max) and the minimum (Min) compared to other tasks in terms of a given PC score. Lower triangular arrows are directed toward the tasks which have higher PC scores, when comparing two tasks. The non-significant (p >0.05) differences are shown by dash signs. The type(s) and frequency band(s) associated with each component (using the features have the strongest impacts; refer to Figure 3C) are reported in the first column*.

*Each element of the sub-matrices is corresponding to the listening tasks labeled above and to the left side: LA, lecture attended, BA, background attended, BUA, background unattended; Each sub-matrix is corresponding to the background noise type: MT, multi-talker; HW, highway; FT, fluctuating traffic and EEG principal component scores (PCS_i_) labeled above and to the left side, respectively*.

**Table 2:**
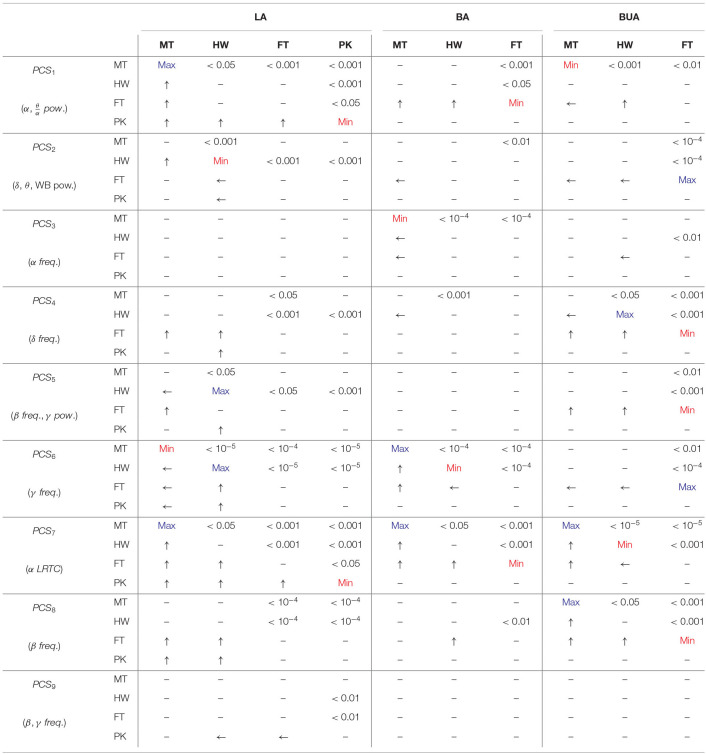
Tukey *post-hoc* multiple comparison testing for within-task model.

*Tukey variable is the background noise type and all possible pairs of means in each subtable are compared. Significant p-values are reported in upper triangular. Data can be decoded like Table 1. The type(s) and frequency band(s) associated with each component (using the features have the strongest impacts; refer to Figure 3C) are reported in the first column*.

*Each element of the sub-matrices is corresponding to the background noise type labeled above and to the left side: MT, multi-talker; HW, highway; FT, fluctuating traffic; Each sub-matrix is corresponding to the listening tasks: LA, lecture attended, BA, background attended, BUA, background unattended and EEG principal component scores (PCS_i_) labeled above and to the left side, respectively*.

In within-background modeling (see Table 1), the first PC score is (significantly) the highest and lowest in the BUA and BA tasks for all the background noises, respectively. Moreover, the BUA task has the highest **PCS**_3_ values compared to other tasks for all background noises. For the MT background noise, the LA has the highest **PCS**_2_ compared to other tasks. The fourth PC score exhibits significant contrast between background attended and other tasks for all background noises. The lecture attended can be discriminated from other tasks for all background noises by the fifth PC score. The sixth PC score has a significant contrast between LA and BA tasks in the MT background noise. The seventh PC score is the highest for the BUA compared to other tasks in the MT and HW noises. For all the background sounds **PCS**_8_ is consistently minimal in the BA task. Finally, the ninth PC score is the maximal and minimal for the BA task in the MT and FT sounds, respectively.

In within-task modeling (see Table 2), the MT has the highest **PCS**_1_ compared to other background noises in the LA task, whereas in the BUA task, the MT has the lowest **PCS**_1_. The second PC score in the HW is significantly lowest compared to other noises in the LA task. The third PC score exhibits only significant differences in the BA and BUA tasks. The background sounds can be discriminated by the fourth PC score in the LA and BA tasks. The fifth PC score has the highest values in the HW noise during the LA task compared to other background noises. The sixth and seventh PC score are significantly able to distinguish the background sounds for all the listening tasks. The eighth PC score exhibits the highest value for the MT and HW in the LA and BA tasks, respectively. The ninth PC score is not very capable of distinguishing the background sounds.

**Remark 1**: The statistical results reported in Tables 1, 2 have been obtained by eliminating the person-dependent effects, while in the previous section, the topographic maps (Figure 4) were obtained by averaging across all subjects without eliminating the person-dependent effects. As a result, the differences are seen in Figure 4 are not only due to differences between tasks and between noises (like Tables 1, 2) but also due to differences between participants. This means that some of the differences seen in the tables and the topographies are not comparable due to the presence of the effect of the changes between individuals. For example, in the highway noise, although the second PC scores of the BUA task are qualitatively lower than other tasks based on Figure 4, Table 1 shows only the dominance of the LA over the BUA. To explain this difference, we performed Tukey's *post-hoc* testing of linear fixed-effect modeling (without **participant** as a random factor). The *post-hoc* test revealed that BUA < BA (*p* < 10^−5^) and BUA < LA (*p* < 10^−5^) meaning that the second PC score can be affected by individual differences likely due to the wideband power (1 − 45 Hz) contributing to this component.

**Remark 2**: Referring to section 2.8, in Tables 1, 2, the results were shown for a model also including the subregions. This implies that a statistically significant difference in one subregion is sufficient for obtaining significant differences. In the maps of Figure 4, the reader is expected to interpret the differences in this way. However, the effect of different subregions were separately investigated to model the exam results in the LA task (refer to section 2.9 and 3.4).

### 3.4. Predictability of Exam Results in Lecture-Attended Task

**Figure 5:**
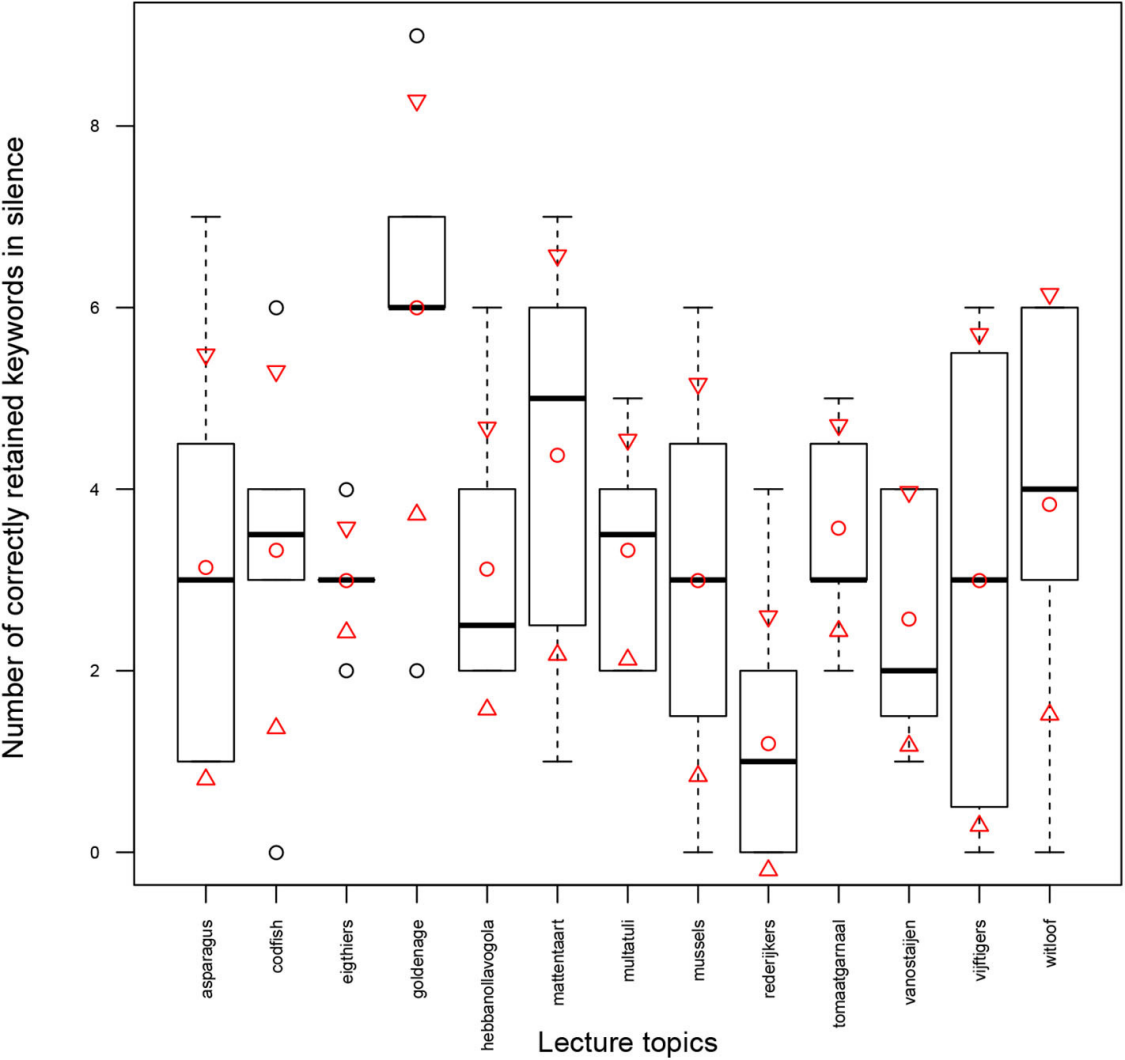
Number of correctly retained keywords in silence (LA-PK) across 13 topics; mean and standard deviation values are shown by circles and triangles, respectively. The box-plots display the median marked as a bold line. The lower and upper whiskers represent another 50% data distributed outside the interquartile box.

As noted in section 2.9, the exam z-score defined in Equation (5) is a fairer measure compared to the exam scores ($\frac{\#\text{Correctly Retained Keywords}}{\#\text{Total Keywords}}$) to quantify the amount of information that participants have actually acquired and retained from the lectures. To normalize the exam results (the number of correctly retained keywords) and find the exam z-scores, the exam results of a lecture-attended task in pink noise (lecture in silence) were used. Figure 5 visualizes the number of correctly retained keywords for lecture attended task in pink noise across thirteen topics. Mean and standard deviation values (μ_*pink*_ and σ_*pink*_ in Equation 5) are shown by circles and triangles, respectively. The boxplots display the median marked as a bold line. The lower and upper whiskers represent another 50% data distributed outside the interquartile box. As can be seen from Figure 5, the number of retained keywords in silence for different topics are different, and hence, the difficulty of retaining information in each topic is different.

In order to assess the effect of background noise type on predicting the exam z-scores, the exam z-scores were modeled using formula (10) and then, Tukey *post-hoc* multiple comparison testing was used to compare the background noise types. The statistical results are reported in Table 3. As can be seen, there are significant differences between pink and multi-talker, between pink and highway, and between fluctuating traffic and multi-talker noises. This means that the exam z-scores are higher in the pink noise (LA in silence) than those of in the multi-talker and highway background noise (as we expected). In addition, the fluctuating traffic background noise leads to the higher exam z-scores compared to those of the multi-talker background noise. Therefore, compared to the fluctuating traffic noise, the multi-talker noise leads to more difficult condition for information retention. Note that pink noise refers to a very low-level pink noise (see section 2.2) and means that subjects have listened to the lectures in silence.

**Table 3:** Effect of background noise on exam z-score; Tukey *post-hoc* multiple comparisons between different types of background noise for modeling exam z-score in lecture attended task (using mixed-effect modeling).

**Background type**	**Pink**	**Highway**	**Multi-talker**	**Fluctuating traffic**
Pink	–	*p* < 0.05	*p* < 0.01	–
Highway	↑	–	–	–
Multi-talker	↑	–	–	*p* < 0.05
Fluctuating traffic	–	–	←	–

*Tukey variable is the background noise type and all possible pairs of means are compared. Upper triangular elements indicate corresponding p-values (p) between two background noises (if p <0.05). Lower triangular arrows are directed toward the background noises which have higher (better) exam z-scores*.

To identify the link between EEG-PC scores and the exam z-scores, both fixed and mixed-effect models were employed as presented in section 2.9. Note that the EEG-PC scores used in this section were obtained by averaging across the channels corresponding to the given subregions. The models were compared using two criteria. First, χ^2^ test was used to compare between the two models using “anova” function in STATS v3.6.2 package of the statistical software R (R Core Team, 2019). A good model not only needs to fit data well—it also needs to be parsimonious. This criterion takes the model objects as arguments and returns an ANOVA testing whether or not the more complex model is significantly better at capturing the data than the simpler mode. If the resulting *p*-value is <0.05, we conclude that the more complex model is significantly better than the simpler model. If the *p*-value is >0.05, we should favor the simpler model.

The second criterion used to compare the fitted models was the Akaike information criterion (AIC) (Akaike, 1974). When comparing models fitted by maximum likelihood to the same data, a lower AIC value indicates a better fit. We have used “extractAIC” function in STATS v3.6.2 package of the statistical software R (R Core Team, 2019). The following equation is used to estimate AIC: −2log(*L*)+(*k* × *edf*), where *k* = 2, *L* refers to the likelihood, and *edf* stands for the equivalent degrees of freedom (i.e., the number of free parameters for the models) of fit.

Table 4A reports the predictability of exam z-scores based on linear fixed-effect modeling (without considering participant as a random factor). The following predictors (fixed factors) were used: (1) no fixed factor (constant), (2) background type, and (3) 54 EEG-PC scores as defined by formulas (6), (7), and (8), respectively. Furthermore, a stepwise fixed-model regression was performed to regress exam z-score using the most significant EEG-PC scores (refer to section 2.9). *P*-values shown on the upper diagonal of Table 4A, suggest that there are pairwise significant differences between all models except between two models which use 54 EEG-PC scores and stepwise EEG-PC scores as the fixed factors. This means that the stepwise model (simpler model) is better than the full model (more complex model) in terms of χ^2^ test criterion.

**Table 4:** Predictability of exam z-scores using **(A)** fixed-effect and **(B)** mixed-effect models.

**(A) Fixed-effect model: [Exam z-score ~ 1 + Fixed Factor]**.				
**Fixed factor**	**Constant**	**Background type**	**54 EEG-PC scores**	**Stepwise EEG-PC scores^a^**
Constant	*AIC* = 134.26	*p* < 0.01	*p* < 10^−12^	*p* < 10^−15^
Background type	←	*AIC* = 128.67	*p* < 10^−10^	*p* < 10^−15^
54 EEG-PC scores	←	←	*AIC* = 89.73	–
Stepwise EEG-PC scores	←	←	←	*AIC* = **38.72**
^a^Contributing PC scores (PCSs):				
*p* < 10^−4^ → Parietal PCS 7 (−0.86);				
*p* < 10^−3^ → Central PCS1 (0.63);				
*p* < 0.01 → Occipital PCS 1 (−0.51), Occipital PCS 2 (−0.33), Occipital PCS 7 (0.50), Occipital PCS 9 (−0.32), Frontal PCS 4 (−0.33), Central PCS 5 (0.57), Central PCS 8 (−0.41), Left Temporal PCS 4 (0.52);				
*p* < 0.05 → Occipital PCS 4 (−0.25), Frontal PCS 3 (−0.16), Parietal PCS 1 (0.42), Parietal PCS 5 (−0.40), Parietal PCS 8 (0.25), Left Temporal PCS 2 (0.65), Left Temporal PCS 1 (−0.53), Left Temporal PCS 6 (0.26);				
^•^*p* < 0.2 → Frontal PCS 6 (−0.14), Frontal PCS 7 (0.14), Left Temporal PCS 3 (0.12), Left Temporal PCS 5 (−0.18), Right Temporal PCS 2 (−0.42).				
**(B) Mixed-effect model: [Exam z-score ~ (**1**|**Participant**) + **1** + Fixed Factor]**.				
**Fixed effects**	**Constant**	**Background type**	**54 EEG-PC scores**	**Stepwise EEG-PC scores**^b^
Constant	*AIC* = 839.75	*p* < 0.01	*p* < 10^−3^	*p* < 10^−7^
Background type	←	*AIC* = 830.94	*p* < 0.01	*p* < 10^−5^
54 EEG-PC scores	←	←	*AIC* = 851.45	–
Stepwise EEG-PC scores	←	←	←	*AIC* = **806.76**
^b^Contributing PCSs:				
*p* < 10^−5^ → Parietal PCS 7 (−0.37)				
*p* < 10^−4^ → Occipital PCS 2 (−0.47);				
*p* < 0.001 → Central PCS 1 (0.68), Left Temporal PCS 2 (0.35);				
*p* < 0.01 → Central PCS 8 (−0.45);				
*p* < 0.05 → Occipital PCS 1 (−0.17), Central (0.39) and Parietal (−0.38) PCS 5, Parietal PCS 4 (−0.12), Parietal PCS 8 (0.37), Left Temporal PCS 1 (−0.41).				

*Upper triangular elements are pairwise p-values (p) when two models are compared using χ^2^ test (if p <0.05). Lower triangular arrows are directed toward the better models when comparing two models. If the resulting p-value is <0.05, the more complex model is significantly better at capturing the data than the simpler model. If the p-value is >0.05, we favor the simpler model. Main diagonal elements indicate AIC values for given models. The lowest AIC value (corresponding to the best model) is shown in bold. The PC scores obtained by the stepwise method are reported below the tables and (•) denotes the regression coefficient (slope) of each factor*.

AIC values shown on the main diagonal of Table 4A, suggest that stepwise EEG-PC scores can predict the exam z-scores better than other models (the lowest AIC). We found the 23 predictors that play more significant roles to predict the exam z-scores. The names of these predictors, their *p*-values (to predict the exam z-scores), and their coefficient (slope in regression) are reported below Table 4A. They were ordered according to their statistical significance. As can be seen from Table 4. the parietal PC score 7 (related to alpha LRTC), which is negatively correlated with exam z-scores, is the most important predictor to model the exam z-scores using the linear fixed-effect modeling.

The results of the mixed-effect models (formulas 9–11) to model exam z-scores are presented in Table 4B. By including the participant as a random factor, the models are less likely to be affected by individual differences. Therefore, those EEG features that contribute to differentiate between participants are expected to be less relevant in this modeling. In contrast to the fixed-effect model, in the mixed-effect model, background noise type can better predict the exam z-scores compared to 54 EEG-PC scores (in terms of AIC and not χ^2^ test). However, the stepwise EEG-PC scores results in a significantly better model than knowing background noise type to predict exam z-scores (the lowest AIC).

According to the tables, in the both fixed and mixed-effect models, the modes which use stepwise EEG-PC scores predict the exam z-scores better than all other models. It is worth noting that unlike the fixed-effect model which all the components in the certain subregions play the significant roles in predicting, in the mixed-effect model, the most contributing predictors are limited to the PCS 1, 2, 4, 5, 7, and 8 in the particular subregions (as can be seen from below Table 4B). These results are consistent with the results of section 3.3, where the importance of these components (especially PCS 7) to distinguish between the background noises in the lecture attended task was shown (refer to Table 2). The relationship between these components and hypotheses presented in the introduction section and their underlying mechanisms will be discussed in the next section.

## 4. Discussion

The present study used a single-trial 64-channel EEG measurement and ecologically valid stimuli to investigate the neural correlates of acquiring and retaining vocally presented information. To identify significant EEG components, a broad set of three listening tasks were performed: (1) attentive listening to 5-min lectures in the environmental sound (LA), (2) attentive listening to environmental sounds (BA), and (3) inattentive listening to environmental sounds (BUA). The environmental sounds included multi-talker, highway, and fluctuating traffic sounds. During this unsupervised learning step, a wide range of features of sensor-space EEG signals were collected and their principal component scores (PCSs) were calculated. Unlike the attention decoding studies that aim to explicitly decode an attended from unattended speech stream based on the supervised approach (Horton et al., 2014; O'Sullivan et al., 2014), we aimed to distinguish between attentive and inattentive listening conditions. To this end, we used an unsupervised learning method that, as such, did not require knowledge of the attended sound signal.

During the LA task, the mixture of verbal lectures and different types of background noise were presented. The lectures were related to topics for which prior knowledge is expected to be minimal. A written exam was taken after the experiment to quantify the amount of information that participants have acquired and retained from the lectures. Since the exam included the questions related to fact and insight, memory is expected to be more specifically involved. It is worth noting the following: (1) although the background sounds could distract the participants while listening to the speech, they did not mask the speech energetically, and (2) no visual distractor was presented during the experiment.

### 4.1. Essential EEG-PC Scores to Predict the Exam Results

The predictability of exam results of the LA task by the EEG-PC scores (EEG-PCSs) has been assessed by linear fixed and mixed-effect modeling of the exam z-scores. It is expected that differences in the exam z-scores can arise from the instantaneous listening state but also from the overall state, personal traits, physiology, and prior knowledge, hence both fixed and mixed-effect models were used to regress the exam z-scores. The fixed-effect model, not considering participant as a random factor, assumes that all relevant differences for predicting exam z-scores are visible in EEG-PCS, whereas the mixed-effect model, considering participant as a random factor, assumes some personal differences are not visible in the EEG-PCS. We first consider the latter approach.

Firstly, it could be confirmed that knowing the type of background sound improves the predictability of exam z-scores (refer to Table 4). Exams on information presented in background noise always gave significantly lower scores, except for fluctuating traffic noise that did not seem to significantly affect exam z-scores (refer to Table 3). Note that in our experiment, noise may affect speech perception, listening comprehension, distraction, and memory encoding. Speech perception in noise was found to be consistently worse in babble than in traffic noise in previous research (Shukla et al., 2018). For episodic memory tasks, it was found that encoding under traffic noise and meaningful irrelevant speech were worse than under silent conditions, but scores were lower for traffic noise than for competing meaningful speech (Hygge et al., 2003). Thus, our results seem to confirm previous works. We can now turn to the question of whether EEG allows us to disentangle the multitude of interacting effects that play a role.

A stepwise mixed-effect model identified that a few specific EEG-PCSs play a more significant role in modeling the exam z-scores (refer to Table 4B). These EEG-PCSs are the central, occipital, and left temporal PCS 1, the occipital and left temporal PCS 2, the parietal PCS 4, the central and parietal PCS 5, the parietal PCS 7, the central, and parietal PCS 8. The underlying mechanisms of these components and their links with our hypotheses are discussed based on the unsupervised learning phase and the previous studies as follows.

- The first component: overall attentive state

In general, the alpha-band activity has been assumed as an idling rhythm (Pfurtscheller et al., 1996) meaning the power of alpha activity increases during resting state and conditions of mental inactivity. During the cognitive effort, alpha activity usually diminishes, which is referred to as alpha desynchronization (Pfurtscheller and Da Silva, 1999; Sauseng et al., 2005). In addition, previous studies have argued increased occipital (task-irrelevant) and decreased frontal (task-relevant) alpha activity can reflect the distracted auditory attention (Pfurtscheller and Da Silva, 1999; Sauseng et al., 2005; Clayton et al., 2015). Our results showed the occipital and PCS 1 is negatively correlated with the exam z-scores (*p* = 0.02, *s* = −0.17, where *s* is the slope of corresponding factors in the linear regression). Based on the results yielded by PCA (refer to Figure 3), the alpha peak power and alpha bandwidth are the most positively and negatively contributing feature to this component, respectively. Therefore, an increase in the exam z-scores can be associated with a decrease in this component score due to overall mind wandering and distracted attention. This statement is in accordance with the unsupervised analysis results where the multi-talker and pink (lecture in silence) PCS 1 is the maximal (the least attention) and minimal (the highest attention) compared to other background sounds during the lecture attended task (see Table 2). In addition, the ratio of theta to alpha power (RPTA in Figure 3) also positively contributes to this component which also confirms that an increase in PCS 1 indicates the deterioration in attention (in agreement with Holm et al., 2009; Borghini et al., 2014).

- The fourth component: low-frequency speech envelope following

The parietal PCS 4 is negatively correlated with the exam z-scores (*p* = 0.027, *s* = −0.12). The fourth PC is strongly determined by various characteristics of the delta frequency band, such as bandwidth, central frequency, and spectral edge frequency (refer to Figure 3). This frequency band is observed during speech envelope following (Kerlin et al., 2010; Ding and Simon, 2014; Vanthornhout et al., 2019). In addition, the gamma central frequency and the alpha-band LRTC negatively contribute to the fourth PCS and are visible in the occipital, temporal, and parietal regions (see Figures 3, 4). The unsupervised analysis revealed that the fourth PCS exhibits the highest and lowest values in background attended and unattended tasks, respectively (see Table 1). Therefore, the fourth PCS may reflect speech envelope following and listening attentively without necessarily linguistic processing or gating out (our third hypothesis). This interpretation could be consistent with the lower values (more negative values) of the parietal and occipital fourth PCS in fluctuating traffic noise compared to other background noises in the lecture attended and background unattended tasks (refer to Figure 4).

- The fifth component: decreased focusing during listening

The parietal fifth PCS exhibits a reverse relationship with the exam z-scores (*p* = 0.020, *s* = −0.38). The positively contributing EEG features to the fifth PCS include the beta central frequency and the gamma absolute power. Based on the unsupervised analysis, the fifth PCS is the lowest in the lecture attended (LA) task compared to other tasks for all background noises (refer to Table 1). Therefore, decreased fifth PCS is likely associated with more focus during listening, where the exam scores are expected to improve as well.

- The sixth component: cognitive prediction error

Although the sixth PCS is not obtained from the mixed-effect stepwise regression as a contributing component, the left temporal PCS 6 is the most significant component obtained from the full mixed model (*p* = 0.02, *s* = 0.55). The sixth PCS positively loads on the gamma spectral edge frequency, bandwidth, and central frequency. Moreover, the frontal and central sixth PCS is negatively correlated with the exam z-scores (*s* = −0.10 and *s* = −0.20). Based on the unsupervised analysis, the sixth PCS is more discriminating between the background noises. Its highest values are observed for attended speech in continuous highway sound (LA-HW) and for attended multi-talker sound (BA-MT) (refer to Table 2). These two conditions have in common that one may rely on linguistic processing and prediction to complete the information. This factor is therefore likely associated with predictive coding. Higher values of the sixth PCS result in lower exam z-scores which may be explained by the fact that a need for prediction to complete the information may result in poor encoding. This finding is in line with Bastos et al. (2012), Sedley et al. (2016), and Alexandrou et al. (2017) where has been shown the prediction violations or errors (our fifth hypothesis) are encoded by gamma-band activity (especially over higher brain areas). It was also found that this component over the left temporal region is positively correlated with the exam z-scores reflecting task-relevant gamma-band activity role on speech processing in alignment with Giraud et al. (2007), Morillon et al. (2012), and Alexandrou et al. (2017).

- The seventh component: alpha-as-inhibition and inhibition-excitation balance

The parietal seventh PCS, which positively loads on alpha-band LRTC, is negatively correlated with the exam z-scores (*p* = 8 × 10^−6^, *s* = −0.37). Interestingly, this PC score in multi-talker noise and independent of task type is significantly dominant compared to other background noises. Increased alpha-band LRTC reflects that the autocorrelations of alpha activity slower decay in power-law behavior and as a result, the self-similarity of alpha activity increases. In fact, high levels of alpha-band LRTC reflect the enduring alpha waves. In agreement with Poil et al. (2012), this increased self-similarity or long-lasting changes could reflect more balance between excitation and inhibition states of alpha-band activity during the auditory stimulus (our second hypothesis). Both excitation and inhibition sates are therefore involved during attentive listening to the lecture in multi-talker sound, which is required for more listening effort due to multi-talker distraction. In contrast to multi-talker, attentive listening to lectures in pink noise (lecture in silence), the alpha-band LRTC is the lowest compared to other noises due to less need for inhibition during listening. In fact, during this listening condition, the excitation state is more dominant than the inhibition state. Increased PCS 7 could thus be associated with a higher inhibition-excitation balance. This component can be linked to the alpha-as-inhibition (Clark, 1996; Uusberg et al., 2013) hypothesis (our first hypothesis) where alpha synchronization reflects suppression of irrelevant information (inhibition).

For the fixed-effect model, where all differences between people are assumed to be explainable through EEG, also adding second (over non-occipital regions), third, sixth, and ninth PCSs improves the predictability of exam results (refer to Table 4A). The second PCS loads strongly on the wide-band absolute power and absolute powers in the low-frequency bands (delta and theta). It is probably related to the observability of EEG for each specific person and may not indicate specific brain-related functions. The third PCS mainly loads on alpha peak frequency, alpha central frequency, and related factors. As PCS 1, the third PCS is significantly higher in the BUA task. Literature is not univocal on the expected trends in relation to tasks (Angelakis et al., 2004; Mierau et al., 2017) but points at a significant difference between persons (Klimesch et al., 1993; Haegens et al., 2014). The latter may explain why PCS 3 only occurs as a significant predictor in the fixed-effect model where it helps to differentiate between persons.

### 4.2. EEG-PC Scores Related to Task Difficulty-Based Cognitive Load

In this experiment, adding background sound to the lectures increases the effort needed to process the sound, but it may also affect cognitive load and task difficulty. The cognitive load of subjects has been assessed from different perspectives using EEG depending on the type of task. For instance, the task difficulty during the intelligence test (Friedman et al., 2019) and learning task (Mills et al., 2017) as the cognitive load has been linked to EEG features. Moreover, the cognitive load during a visual task has been associated with the attentional demand using an ERP analysis (Grassini et al., 2019). There is no unique EEG feature that is directly related to cognitive load. Theta power has been suggested as an indicator for the average cognitive load of subjects and the linguistic complexity of educational videos (Castro-Meneses et al., 2019). Mu rhythm oscillations (8 − 13 Hz over the sensorimotor cortex) could be affected by the cognitive load during speech perception due to attention and working memory processes (Jenson et al., 2019). In addition to the task difficulty, the listener's skill also may affect the cognitive load.

In this paper, although the cognitive load of listeners has not been explicitly investigated, some PCSs may reflect the task difficulty-based cognitive load, such as the sixth and seventh PCSs (reflecting the prediction error and the inhibition during listening, respectively). However, caution is needed to link neural results to these behavioral outcomes as this study is based on a sample of young adults only. Aging populations might react differently.

Since there are more noiseless gaps during fluctuating traffic sound compared to the highway sound (refer to Figure 2), it is expected that less mental resources are needed to predict the missing part (less PCS 6) during LA in fluctuating traffic sound. Therefore, LA in the highway sound (LA-HW) is likely more difficult task compared to LA in the fluctuating traffic sound (LA-FT). However, the task difficulty can be reflected either in the continuous inhibition by increased PCS7 (highway sound) or in the fluctuating inhibition by decreased PCS7 (fluctuating traffic sound). Moreover, in the BUA task, the fluctuating traffic sound is the most difficult sound to predict (the highest PCS6) compared to the other sounds. Although the BUA in the multi-talker sound exhibits more inhibition compared to the fluctuating traffic (higher PCS7), the multi-talker sound in the BUA can be easier predicted (lower PCS 6) compared to the fluctuating traffic sound. These findings may explain the impacts of different types of environmental sound during daily activities.

## 5. Conclusion

The current study showed that it is possible to predict beyond the chance level the amount of vocal information that participants acquire and retain from the lectures presented in different environmental sounds using 64-channel EEG. Five principal component scores of the EEG features obtained under different listening conditions and for different persons were essential for this prediction. Based on their loading on the spectral range and their ability to distinguish between listening tasks, we associate them with overall attentive state, speech envelope following (listening attentively without necessarily linguistic processing), focusing during listening, cognitive prediction error, and specific inhibition. Part of the variance between persons could further be explained by principal component scores that tend to relate to overall signal strength, an indication of observability of EEG signals, and person identification through inter-individual differences between typical alpha peak frequencies.

Inhibition-excitation balance (reflected by alpha-band representation) and predictive mechanisms (reflected by gamma-band representation) play a more important role than might have been expected and could be observed via EEG. Furthermore, the results of comparing the principal components scores of three different auditory tasks (attentive listening to the lecture in environmental noise, attentive listening to the environmental sound, and inattentive listening to the environmental sound) showed the extracted principal components scores are able to discriminate the different listening tasks and background noises. Specifically, (i) the sixth and seventh principal component scores, which reflect prediction error and inhibition-excitation balance, respectively, allow us to distinguish different types of background sound. Moreover, (ii) the type of listening tasks could be completely distinguished by the first and fifth principal component scores, which reflect the overall attentive state and decreased focusing, respectively.

In terms of methodology, by combining different listening conditions to train in an unsupervised way the definition of orthogonal features based on EEG, a more efficient supervised model for the prediction of the memorization of information could be obtained. This methodology could be relevant for assessing the impact of environmental sounds on daily activities, such as communicating, learning, and relaxing as some of the principal components identified could be related to increased cognitive load. They could also be relevant for future artificial intelligence communicating optimally with humans based on observed brain activity. The methodology also allows us to assess individual differences in the ability to process speech in noise.

## Data Availability Statement

The datasets presented in this article are not readily available because further analysis is ongoing. Requests to access the datasets should be directed to the first author. The Matlab® and R® codes implementing the algorithms and statistical analyses are publicly accessible on GitHub (https://github.com/EhsanEqlimi/EEG-Correlates-of-Learning-From-Speech-Presented-in-Environmental-Noise).

## Ethics Statement

The studies involving human participants were reviewed and approved by International Laboratory for Brain, Music and Sound Research (BRAMS), Montreal, Canada. The participants provided their written informed consent to participate in this study. Written informed consent was obtained from the individual(s) for the publication of any potentially identifiable images or data included in this article.

## Author Contributions

EE carried out the data analysis and interpretation, signal processing, statistical analysis, and writing of the manuscript. AB carried out the data acquisition, the experiment design, study idea, statistical analysis, data interpretation, and editing of the manuscript. BD carried out the data interpretation and the editing the manuscript. MS carried out the experiment design, data acquisition and interpretation, and editing of the manuscript. DT carried out the data interpretation and editing of the manuscript. DB carried out the original idea for study, data interpretation, experiment design, and editing of the manuscript. All authors contributed to the article and approved the submitted version.

## Conflict of Interest

BD was employed by company ASAsense. The remaining authors declare that the research was conducted in the absence of any commercial or financial relationships that could be construed as a potential conflict of interest.
